# Supplementary material for: Sphingolipids associate with the chlamydial nucleoid and mark developmental transitions in Chlamydia trachomatis
Source: Nat Commun. 2026 Sep 30;17:10367. doi: 10.1038/s41467-026-77974-3 (PMC13627705; doi:10.1038/s41467-026-77974-3)
Supplement: Supplementary file 1 [file 41467_2026_77974_MOESM1_ESM.pdf]

Sphingolipids associate with the chlamydial nucleoid and mark developmental transitions in *Chlamydia trachomatis*

Marcel Rühling[1§+], Fabienne Wagner[1+], Till Epprecht[2], Paul F. Köhling[1], Fabian Schumacher[3], Stefan Sachs[4], Louise Kersting[5], Julian Fink[5], Lina Girndt[5], Burkhard Kleuser[3], Markus Sauer[4], Jürgen Seibel[5], Gregor L. Weiss[2] and Thomas Rudel[1*]*

[1]Chair of Microbiology, Julius-Maximilians-University Würzburg, Theodor-Boveri-Weg, 97074 Würzburg, Germany

[2]Institute of Medical Microbiology, University of Zürich, Gloriastrasse 28/30, 8006 Zürich, Switzerland

[3]Institute of Pharmacy, Freie Universität Berlin, Königin-Luise-Straße 2+4, 14195 Berlin, Germany

[4]Chair of Biotechnology & Biophysics, Biocenter, Julius-Maximilians-University Würzburg, Am Hubland, 97074 Würzburg, Germany

[5]Institute of Organic Chemistry, Julius-Maximilians-University Würzburg, Am Hubland, 97074 Würzburg, Germany

[§]Current address: MRC Laboratory of Molecular Biology, Francis Crick Avenue, Cambridge Biomedical Campus, Cambridge CB2 0QH, UK.

[+]These authors contributed equally.

*Corresponding author: marcel.ruehling@uni-wuerzburg.de, thomas.rudel@uni-wuerzburg.de

Supplementary Figures

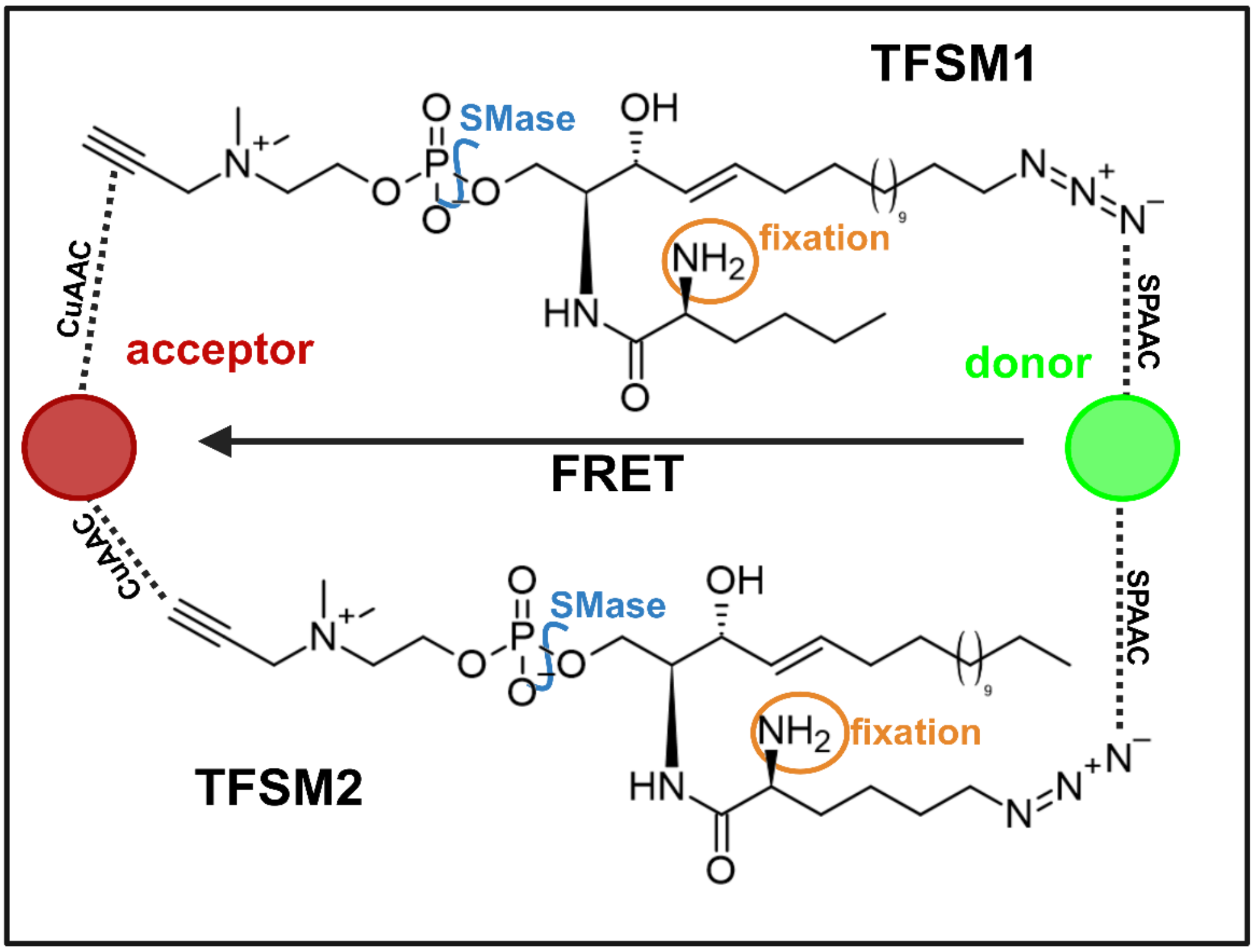

Supp. Fig. 1 Principle of trifunctional sphingomyelin derivatives.
Compared to naturally occurring SM, TFSMs possess an additional amino function (orange) for fixation and linkage into a swellable hydrogel for ExM. Both derivatives possess an alkyne in their head group, which can be modified with a fluorophore (red, acceptor) by copper catalysed azide-alkyne cycloaddition (CuAAC). TFSM1 harbours an azido function in the sphingoid backbone, TFSM2 in the fatty acid chain. Azido functions can be equipped with a fluorophore (green, donor) via strain-promoted azide-alkyne cycloaddition (SPAAC). Fluorophores clicked to the head group and the backbone can interact via Förster resonance energy transfer (FRET). FRET can be used as readout for metabolization of the molecules, since the FRET system is destroyed upon cleavage by an SMase (blue). *Created in BioRender. Rühling, M. (2026) https://BioRender.com/vrmpja7.*

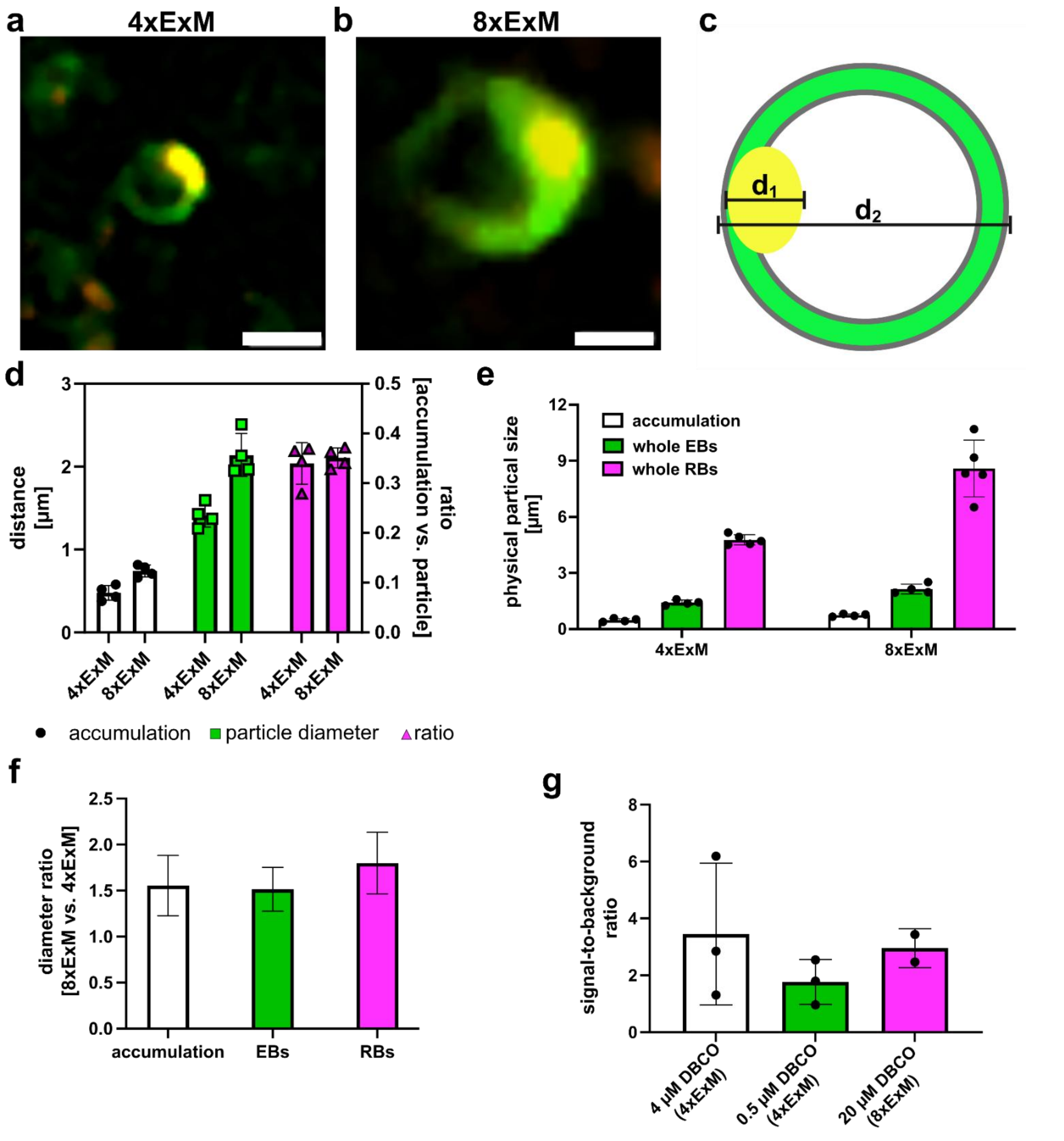

Supp. Fig. 2 Lipid accumulation in EBs is not due to expansion or staining artifacts.
HeLa 229 cells were infected with *C. trachomatis* in the presence or absence of TFSM. Samples were visualized by 4x or 8x ExM as indicated. **a-d, uniform expansion of EBs and their lipid accumulation.** Expansion of EBs upon 4x ExM (a) and 8x ExM (b) was compared. Therefore, the diameter of the lipid accumulation (d_1) and whole EBs (d_2) were measured, and ratios "accumulation vs. particle" were calculated (c, d). n =4. Scale bar: 1 µm (~250 nm with 4-fold expansion factor [a] or ~125 nm with 8-fold expansion factor [b]). **e, f, size comparison of bacterial particles upon 4x and 8x expansion.** Average diameters of RBs, EBs and the EB-associated lipid accumulation were measured in 4x and 8x expanded samples (e) and ratios "8x ExM vs. 4x ExM" of these structures were calculated. Standard deviation was calculated by Gaussian propagation of uncertainty from standard deviation determined from particle diameter measurements used for calculating the ratio. n=4 (accumulation and EBs), n=5 (RBs). (f). **g, determining signal-to-background ratios in lipid accumulations.** Fluorescence mean intensity ($mean_{FL}$) in lipid accumulations was measured in samples incubated with and without TFSM (e). Signal-to-background ratios were determined by [$mean_{FL}$(+TFSM) - $mean_{FL}$(-TFSM)]/ $mean_{FL}$(-TFSM)]. n=3 (4 µM and 0.5 µM) and n= 2 (20 µM). Data are presented as mean values +/- standard deviation. *Panel c was created in BioRender. Rühling, M. (2026) https://BioRender.com/ougrosh.*

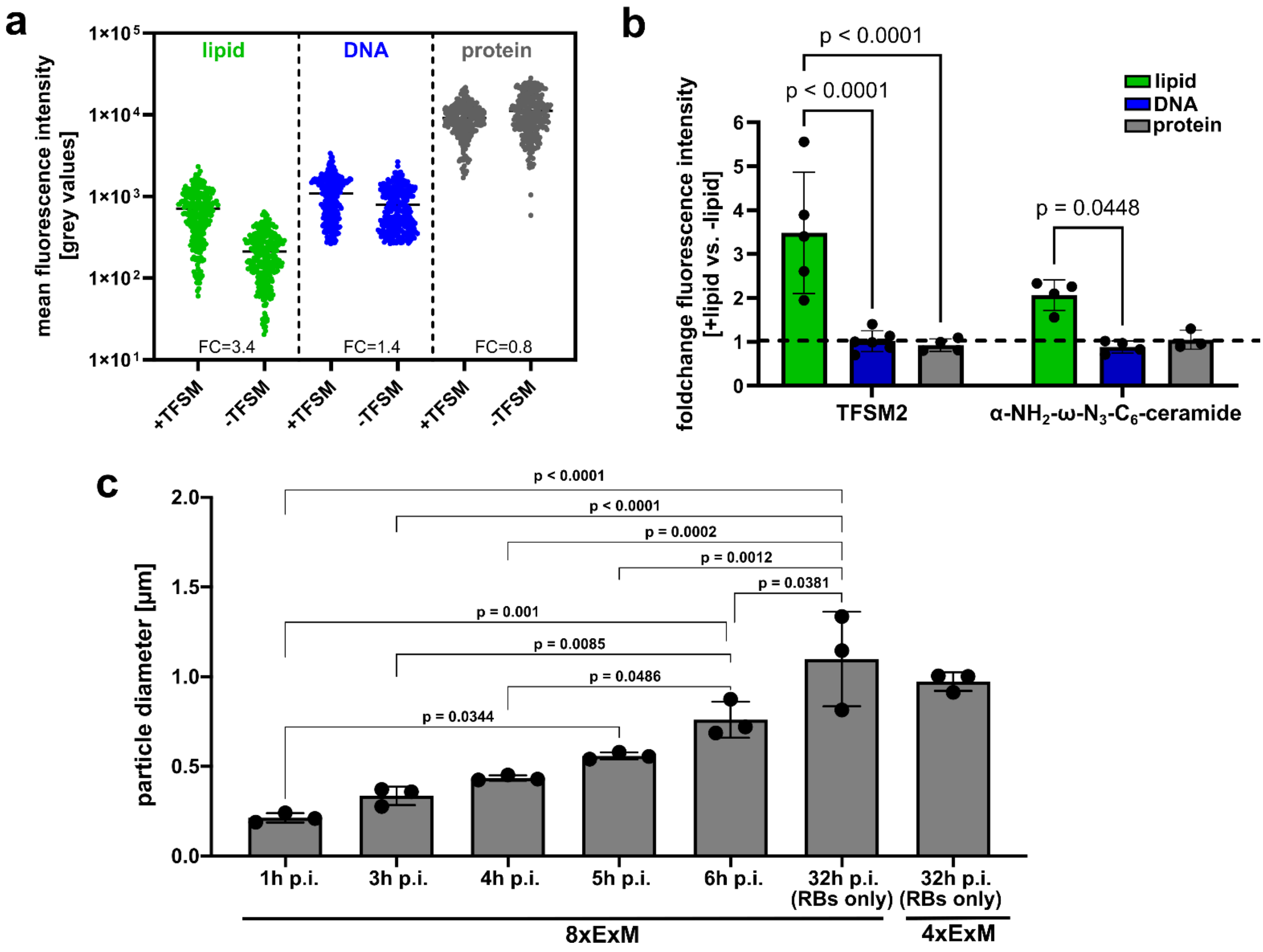

Supp. Fig. 3 TFSM2 and α-NH_2-ω-N_3-C_6-ceramide are enriched in chlamydial nucleoids

a, b, quantification of TFSM2 and α-NH_2-ω-N_3-C_6-ceramide in chlamydial nucleoids. HeLa 229 cells were infected with *C. trachomatis* in the presence or absence of TFSM2 or α-NH_2-ω-N_3-C_6-ceramide and imaged by 8x ExM. Regions containing EB nucleoids were selected in the Hoechst channel and fluorescence mean intensity in the lipid, the NHS ester and Hoechst channel were measured (a). The average fluorescence intensity in all detected nucleoids was determined in lipid, DNA and protein channels. Then the foldchange between samples infected in presence (+lipid) or in absence (-lipid) of the lipid analogues was determined in the respective channels. n=5 (TFSM2) or n=4 (α-NH_2-ω-N_3-C_6-ceramide). **c, particle size of intracellular bacteria continuously increases in the first 6 h of infection.** HeLa229 cells were infected in presence of TFSM, fixed at the indicated time points and visualized by 8x or 4x ExM. Particle diameters of intracellular bacteria were measured and corrected for the respective expansion factors (divided by 4 or 8). For 32 h p.i., only RBs (see Figure 5 for particle classification) were used for the measurement. n=3. Statistics: Mixed-effects analysis (b) or one-way ANOVA (c) and Tukey's multiple comparison. Data are presented as mean values +/- standard deviation.

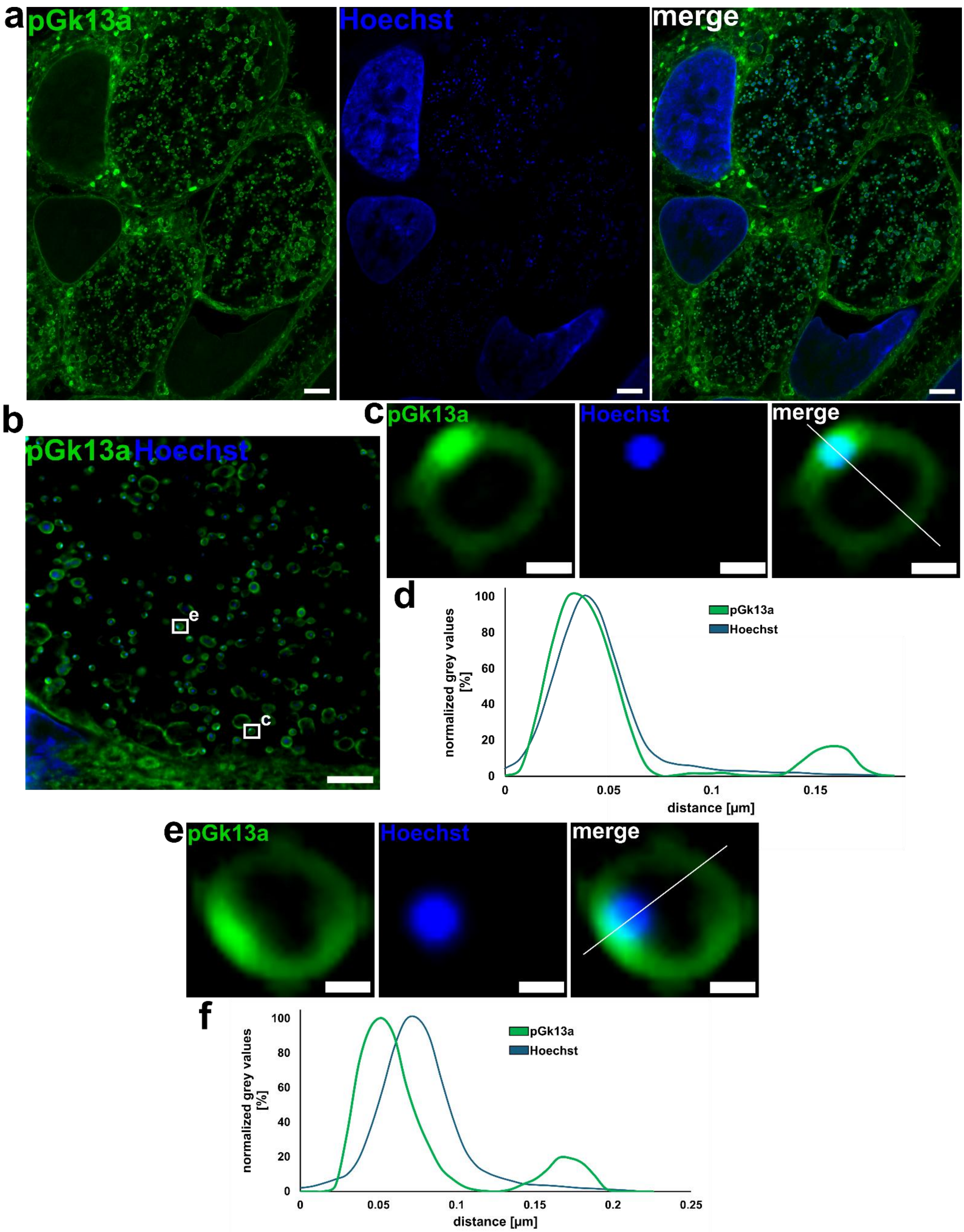

Supp. Fig. 4 The EB nucleoid is in close association with the membrane accumulation.
HeLa 229 cells were infected with *C. trachomatis* for 32 h, fixed, stained with pGk13a and 8-fold expanded. DNA was stained with Hoechst (a, b, c, e). Plot profiles of two individual EBs (c and e) were measured for pGk13a and Hoechst (d, f). Distances in plots were corrected for the expansion factor. Scale bars: a: 16 µm (~2 µm with 8-fold expansion factor), b: 8 µm (~0.5 µm with 8-fold expansion factor), c, e: 0.4 µm (~ 50 nm with 8-fold expansion factor). n=1.

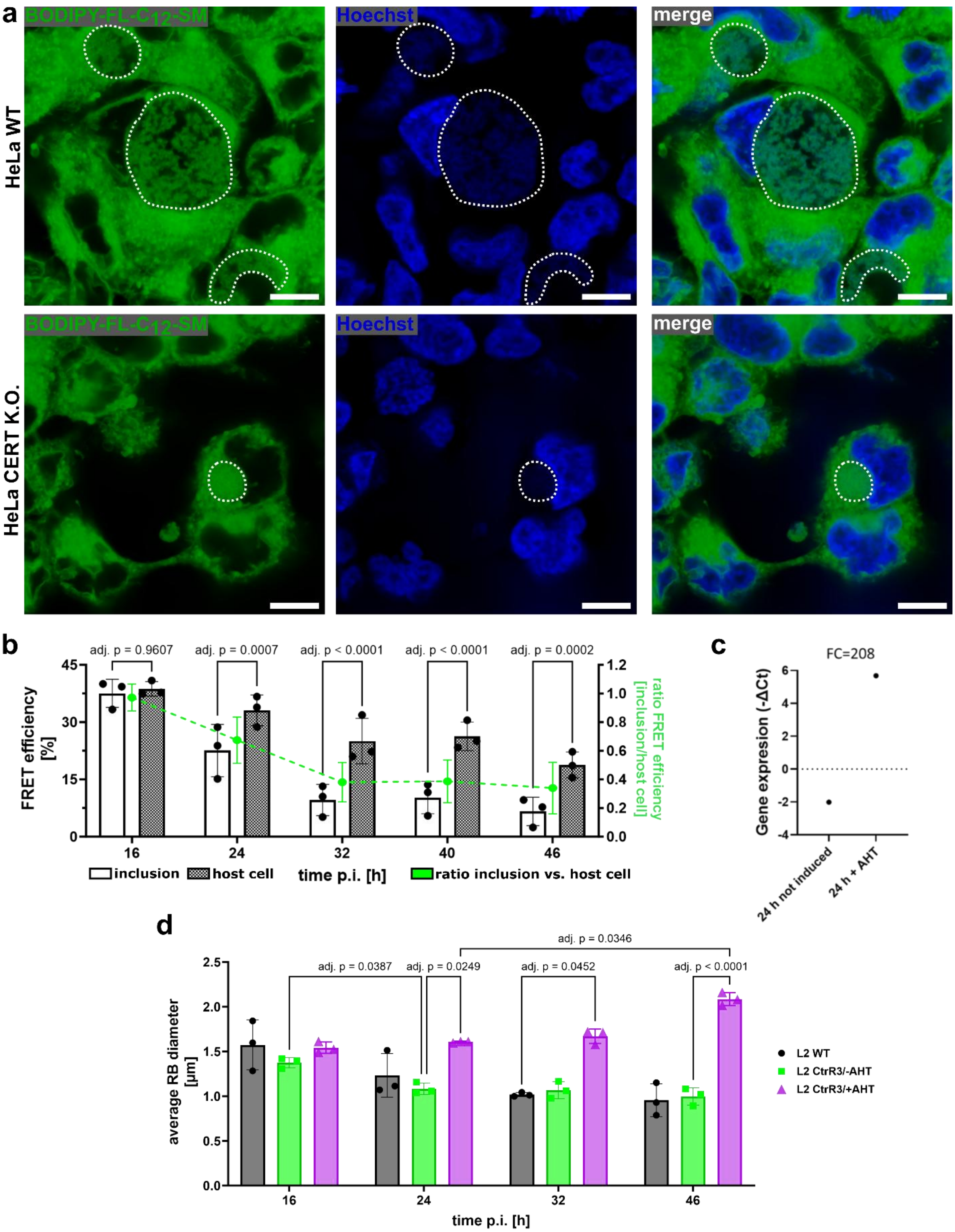

Supp. Fig. 5 Measurement of TFSM metabolization in 4x ExM samples and characterization of the *C. trachomatis* L2 CtrR3 stain.

a, BODIPY-FL-C$_{12}$-SM is taken up by inclusions. HeLa cells were infected with *C. trachomatis*. After 3 h, cells were treated with 10 µM BODIPY-FL-C$_{12}$-SM and fixed 40 h p.i.. DNA was stained by Hoechst. Inclusions are indicated with white lines. n=1. Scale bar: 10 µm. **b, TFSM degradation in inclusions can be measured by 4x ExM.** HeLa 229 cells were infected with *C. trachomatis* in presence of TFSM2 for indicated time points and 4-fold expanded. FRET efficiency in inclusions and host areas were determined by FRET acceptor bleaching. FRET efficiency ratios (inclusions vs. host cell) were calculated (green). n=3. **c, CtrR3 is overexpressed under control of anhydrous tetracycline (AHT).** HeLa 229 cells were infected with the *C. trachomatis* CtrR3 strain in presence or absence of AHT for 24 h. CtrR3 expression was determined by RT-qPCR. n=1. **d, overexpression of CtrR3 affects the size of RBs.** HeLa 229 cells were infected with *C. trachomatis* L2 WT, or the CtrR3 strain either in absence or presence of AHT for the indicated time points. RBs

were visualized by TFSM, and diameters were determined by 4x ExM. n=3. Statistics: Two-way ANOVA and Šidák's multiple comparison (a) or Tukey's multiple comparison (c). Data are presented as mean values +/- standard deviation.

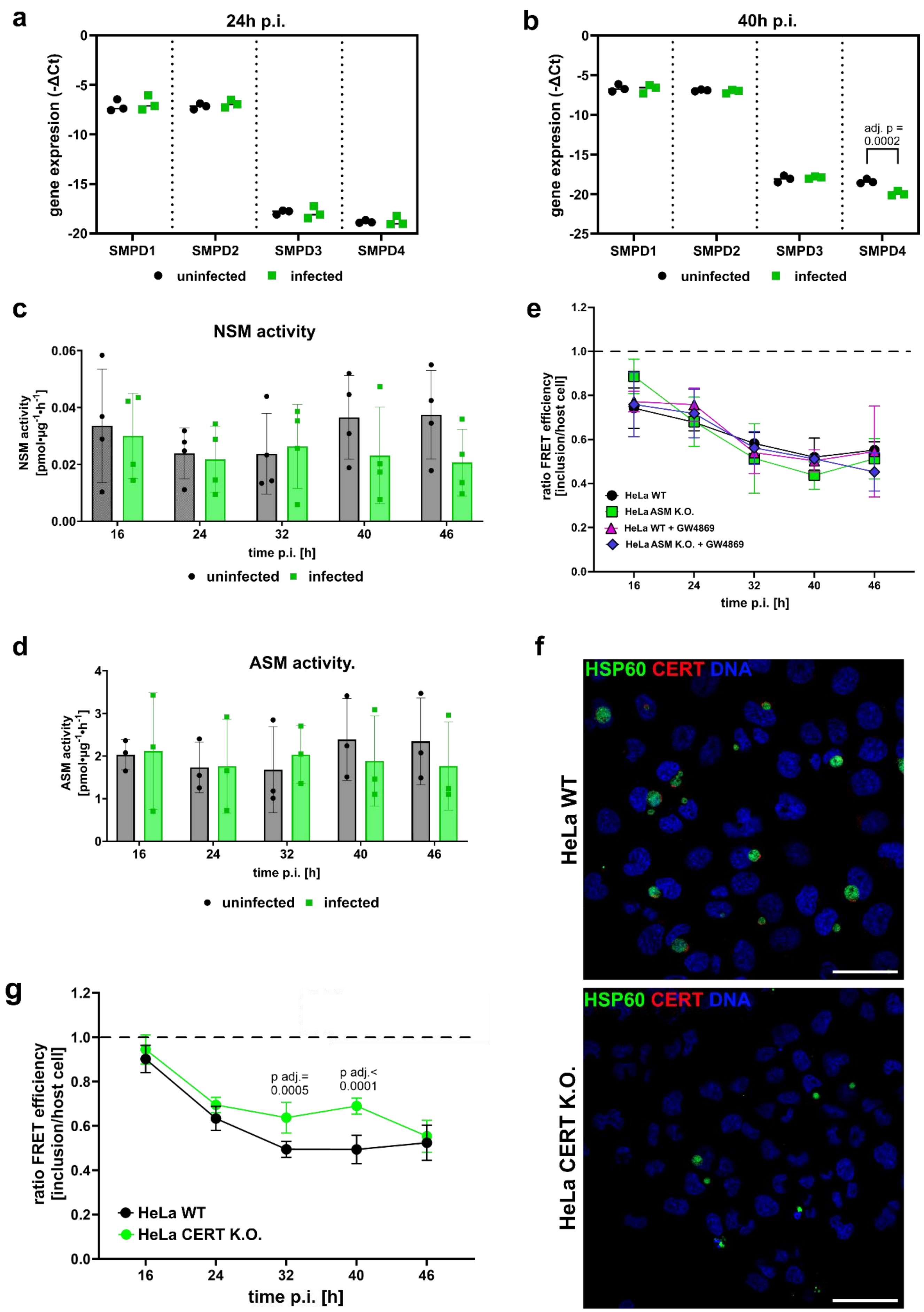

Supp. Fig. 6 Accumulation of metabolized TFSM in inclusions is independent of major host sphingolipid metabolic factors.

a-d, host SMase expression levels and cellular SMase activity is not affected by *C. trachomatis* infection. HeLa 229 WT cells were infected with *C. trachomatis*. At the indicated time point, either RNA was extracted and expression of host cell SMases (SMPD1-SMPD4) were measured by RT-qPCR (a, b) or cells were lysed and cellular neutral sphingomyelinase (NSM, c) as well as acid sphingomyelinase (ASM, d) activity was measured. n=3 (a, b and d). n=4 (c). **e, accumulation of metabolized TFSM in inclusions is independent from host ASM and NSM.** HeLa WT or HeLa ASM K.O. cells were infected with *C. trachomatis* and if indicated treated with the NSM inhibitor GW4869 in presence of TFSM2. At the indicated time point, samples were fixed, TFSM was stained with BODIPY-FL-DBCO and AZDye546-azide, 4-fold expanded and FRET efficiencies in inclusions and host cell areas were determined. FRET efficiency ratios “inclusion vs. host cell” were calculated. n=3. **f, g, absence of CERT impairs infection establishment but does not affect the accumulation of metabolized TFSM in inclusions.** HeLa WT or HeLa CERT K.O. cells were infected with *C. trachomatis* in presence of TFSM2. At the indicated time point, samples were fixed, and either immunostained for chlamydial HSP60, CERT and DNA (e) or stained for TFSM2, 4-fold expanded and FRET efficiency ratios “inclusion vs. host cell” were determined (g). Scale bar: 50 µm. n=4 (g) and n=1 (f). Statistics: Two-way RM ANOVA and Šidák’s multiple comparison. Data are presented as mean values +/- standard deviation.

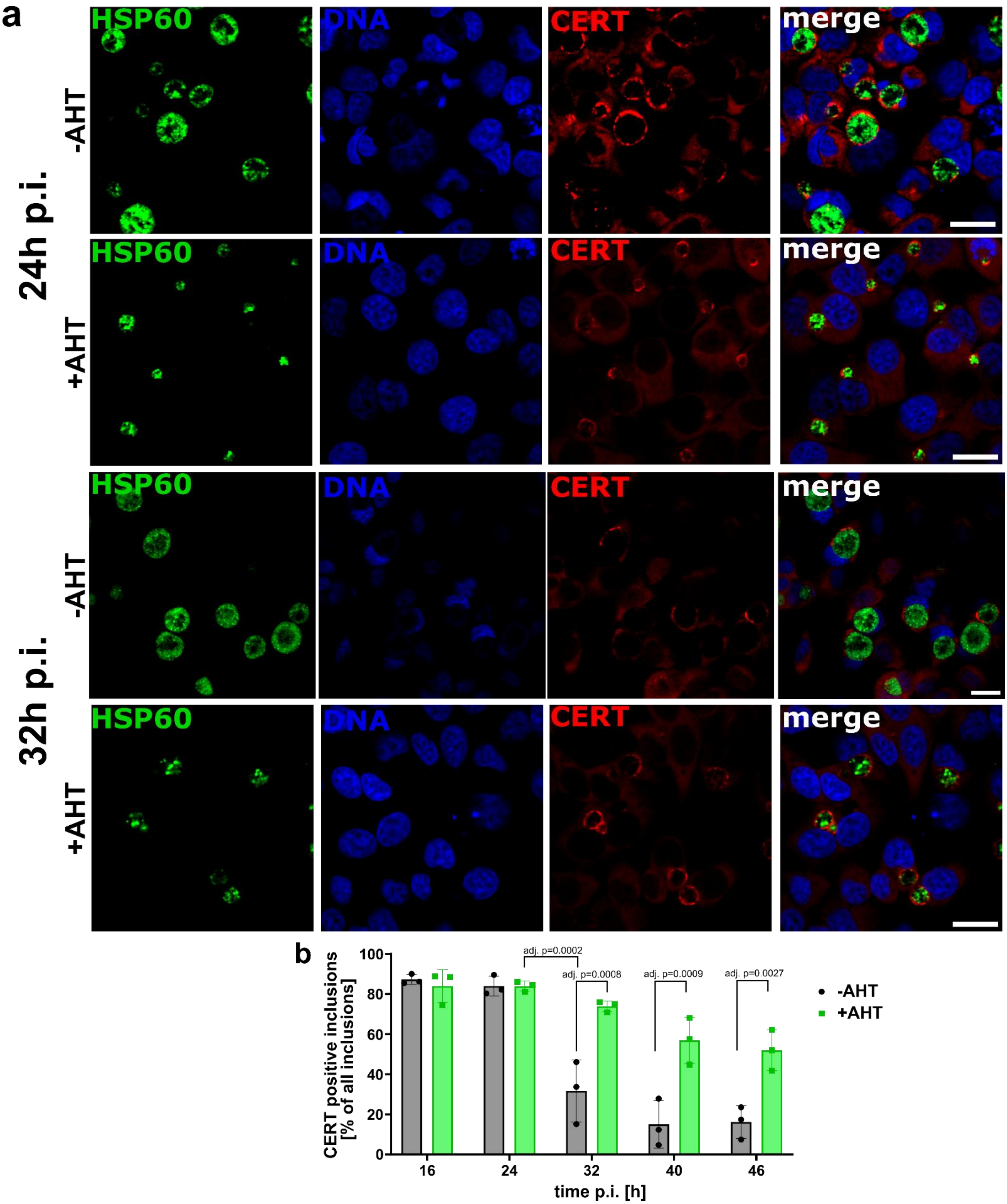

Supp. Fig. 7 CERT is recruited to inclusions of *C. trachomatis* overexpressing the sRNA CtrR3.
HeLa 229 were infected with *C. trachomatis* L2 WT or the L2 CtrR3 strain either in presence or absence of AHT for the indicated duration. Cells were fixed, stained with an anti-CERT (red) as well as an anti-chlamydial HSP60 (green) antibody and recorded by conventional confocal microscopy (a). The proportion of CERT-positive inclusions was determined (b). Scale bars: 20 µm. n=3. Statistics: Two-way RM ANOVA and Tukey's multiple comparison. Data are presented as mean values +/- standard deviation.

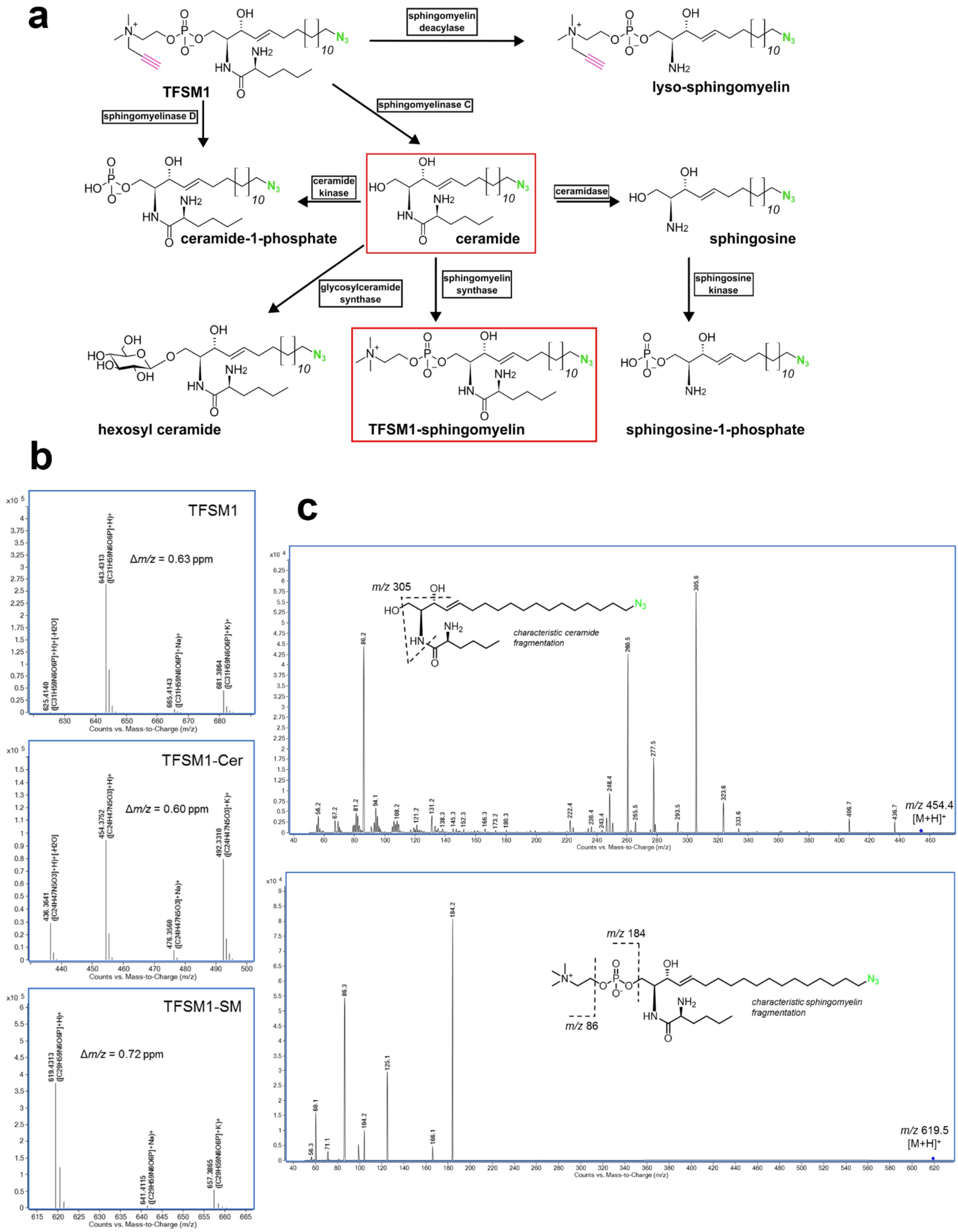

Supp. Fig. 8 Screening for TFSM metabolites during *C. trachomatis* infection.

a, HeLa 229 cells were treated with TFSM1 and either infected with *C. trachomatis* in the presence of TFSM1 or left uninfected. After 24 h or 40 h p.i. lipids were extracted and analyzed for the indicated TFSM1 metabolites. Detected metabolites were highlighted in red. **b,** Identification of the assay substrate TFSM1 and its metabolites TFSM1-Cer and

TFSM1-SM based on the accurate masses (*m/z*) of molecular ions determined by high-resolution QTOF mass spectrometry. **c,** Representative product ion (MS/MS) spectra of TFSM1-Cer (top, recorded with 20 eV collision energy) and TFSM1-SM (bottom, recorded with 60 eV collision energy), which exhibit characteristic MS/MS fragmentation patterns of ceramide and sphingomyelin species, respectively (illustrated with the help of the structural formula as insets).

Supplementary Notes

Supplementary Note 1

TFSM1 and TFSM2 differ by the positioning of the azido function (**Supp. Fig. 1**). While the azido function in TFSM1 is located in the sphingoid backbone in TFSM2, it can be found in the acyl chain. Both molecules enable monitoring of SM metabolization by microscopy and lipidomics. TFSM1 is metabolized slower than TFSM2 in HEK cells.[1] In general, both compounds behaved similar during *C. trachomatis* infection.[1-3] However, dependent on the application the positioning of the azido function can be important.

TFSM1 is ideal for monitoring sphingolipid metabolization by lipidomics as the azido function in the sphingoid backbone can be used to detect the TFSM1-derived sphingosine metabolite when the acyl chain is cleaved off, e.g. by ceramidases (see **Supp. Fig. 8**). In contrast, TFSM2-derived sphingosine is indistinguishable from naturally occurring sphingosine.

Staining TFSM2 with DBCO dyes may be more efficient than TFSM1 as the azido function might be more accessible for the dye when positioned on the shorter acyl chain. Hence, we prefer using TFSM2 when monitoring sphingolipid distribution via 8x ExM.

In theory, FRET originating from TFSM2 can only be detected when the ceramide tail and the head group is present in the same molecule. By contrast, TFSM1 that was processed to lyso-sphingomyelin by a sphingomyelin deacylase can still result in a FRET signal (see **Supp. Fig. 8**).

Supplementary Note 2

To exclude that the accumulation is caused by anisotropic sample expansion, we compared EBs recorded by 4x and 8x ExM and stained with TFSM2 (**Supp. Fig. 2**, a, b). We determined the diameter of the accumulation and of whole EBs and calculated the ratio (**Supp. Fig. 2**, c). The relation between accumulation and EB diameter was similar in 4-fold and 8-fold expanded samples, indicating isotropic sample expansion and excluding expansion artifacts (**Supp. Fig. 2**, d). We also compared the diameter of the accumulation, whole EBs and RBs in 4x- and 8x-expanded samples (**Supp. Fig. 2**, e). In theory, it is expected that structures appear 2-fold larger in 8x ExM compared to 4x ExM images. However, the average accumulations diameter increased by ~1.6-fold, EB diameter by ~1.5-fold and RB diameters by ~1.8-fold (**Supp. Fig. 2**, f). The slight differences in expansion of different structures are statistically not significant, indicating that the sample expands largely isotropically.

The click dyes that we use to label TFSM2 can introduce comparably high background staining. To measure the contribution of this background to the detected signal, we infected samples with *C. trachomatis* in presence or absence of TFSM2. Then, we stained the samples with varying concentrations of the click dye and performed 4x or 8x ExM. We compared the fluorescence intensity within the accumulation in samples incubated with or without TFSM2 and determined the signal-to-background ratio (**Supp. Fig. 2**, g). For dye concentrations commonly used in our expansion protocols, an average signal-to-background ratio of ~3:1 was determined, demonstrating that the accumulation truly contains TFSM2.

Supplementary Note 3

For TFSM2 staining, HeLa 229 cells were infected with *C. trachomatis* in presence or absence of TFSM2 and samples were fixed at 32 h p.i..

For staining with α-NH_2-ω-N_3-C_6-ceramide, cells were infected with *C. trachomatis* for 31 h and then α-NH_2-ω-N_3-C_6-ceramide was added for 1 h before samples were fixed.

Functionalized lipids were stained with BODIPY-FL-DBCO and samples were expanded 8-fold. DNA and proteins were visualized by Hoechst and NHS ester staining. For measuring lipid content in chlamydial nucleoids, areas containing nucleoids were selected based on Hoechst signal and the signal intensities in all three channels were measured. Mean fluorescence intensities measured in individual nucleoids were plotted (see **Supp. Fig. 3**, a for one representative replicate) and the average fluorescence intensities of several independent biological replicates were determined. Ratios between results obtained from samples incubated with the lipid derivatives and samples without added analogs (but stained with the DBCO dye) were

determined (**Supp. Fig. 3**, b). We detected a significantly higher BODIPY-FL signal in nucleoids recorded in samples incubated with the functionalized lipids compared to staining controls.
TFSM2 is taken up by host cell via endocytosis, due to its polar head group. By contrast, α-NH_2-ω-N_3-C_6-ceramide is more rapidly incorporated into cellular membranes. Hence, shorter incubation times are required for α-NH_2-ω-N_3-C_6-ceramide (1 h) compared to TFSM (several hours).

Supplementary Note 4
RBs of WT and the uninduced *C. trachomatis* L2 CtrR3 strain possessed the highest diameter at 16 h p.i. (~1.5 µm) and shrunk during infection to an average size of ~1 µm (**Supp. Fig. 5**, d). By contrast, the diameter of RBs upon CtrR3 overexpression remained at their initial size up to 32 h p.i. and even increased to ~2 µm at 40 h p.i., suggesting a connection between RB-EB redifferentiation and RB size as described previously.[4]

Supplementary Note 5
Since *C. trachomatis* often modulates host cell metabolism for adequate nutrient supply,[5] we hypothesized that TFSM might be metabolized by host cell enzymes and is then shuttled into the inclusion. Human cells possess three neutral (NSM1-3)[6] and one acid SMases (ASM)[7], which catalyze the metabolization of SM to ceramide. We neither detected increased expression levels of these enzymes (**Supp. Fig. 6**, a and b), nor did we measure an increased cellular NSM (**Supp. Fig. 6**, c), or ASM (**Supp. Fig. 6**, d) activity upon infection, suggesting that host cell SMases are negligible for the enrichment of metabolized TFSMs in chlamydial inclusions.
The ceramide transfer protein (CERT) was previously shown to be recruited to chlamydial inclusions[8], which could cause the enrichment of metabolized TFSM within inclusion. To this end, we infected a HeLa CERT K.O. cell line[9] with *C. trachomatis* and again performed FRET acceptor bleaching to determine native TFSM content in inclusions and host cell membranes. Infection rates were strikingly lower in CERT K.O.s compared to the wild type cells (**Supp. Fig. 6**, e). However, metabolized TFSMs accumulated in inclusions despite the absence of CERT, even though in an attenuated fashion (**Supp. Fig. 6**, f).
We also compared recruitment of CERT to inclusions in cells infected with *C. trachomatis* L2 CtrR3 in presence or absence of AHT. Consistently, CtrR3 expression, which strongly attenuated the enrichment of metabolized TFSMs in inclusions (**Fig.5**, e), did not interfere with the recruitment of CERT (**Supp. Fig. 7**, a and b). A large proportion of inclusions were positive for CERT at 16 and 24 h p.i. independently from CtrR3 overexpression. Interestingly, the proportion of CERT-positive inclusions decreased in samples without CtrR3 expression at 32h p.i., whereas most inclusions remained CERT-positive if CtrR3 was overexpressed.
In summary, our data indicates that TFSM metabolization during *C. trachomatis* infection is independent from central factors of host cell sphingolipid metabolism.

Supplementary Methods

Details on chromatography, MS detection and metabolite identification

HPLC settings:

Instrument: 1290 Infinity II (Agilent Technologies)

Column: Poroshell 120 EC-C8 (3.0 × 150 mm, 2.7 µm; Agilent Technologies)

Pre-column: Poroshell 120 EC-C8 (3.0 × 5 mm, 2.7 µm; Agilent Technologies)

Injection volume: 10 µL

Temperature of column compartment: 50 °C

Solvent A: water/methanol (8:2, v:v), 0.1 % formic acid, 10 mM ammonium formate

Solvent B: isopropanol/methanol (9:1, v:v), 0.1 % formic acid, 10 mM ammonium formate

Gradient:

Time (min)	% B	Flow rate (mL/min)
0.00	55	0.25
20.00	80	0.25
22.00	100	0.25
25.00	100	0.25
25.01	55	0.25
33.00	55	0.25

Ion source settings:

Type: Dual Agilent Jet Stream ESI

Polarity: Positive

Gas temperature: 240 °C

Gas flow: 18 L/min of nitrogen

Nebulizer: 20 psi

Sheath gas temperature: 375 °C

Sheath gas flow: 12 L/min of nitrogen

Capillary voltage: 4000 V

Nozzle voltage: 2000 V

Reference masses: *m/z* 121.0509 and *m/z* 922.0098

MS settings:

Instrument: 6550 Quadrupole Time-Of-Flight MS (Agilent Technologies)

Acquisition mode: Full Scan

Mass range: *m/z* 50 – 1000

Scan rate: 3 spectra/s

Details on identified metabolites

Metabolite	Chemical formula	Type	Retention time (min)	*m/z*
TFSM1	$C_{31}H_{59}N_6O_6P$	Analyte	4.33	643.431
TFSM1-Cer	$C_{24}H_{47}N_5O_3$	Analyte	4.68	454.375
TFSM1-SM	$C_{29}H_{59}N_6O_6P$	Analyte	4.29	619.431
C16 SM	$C_{39}H_{79}N_2O_6P$	Analyte	15.09	703.575
C16 Cer	$C_{34}H_{67}NO_3$	Analyte	17.36	538.519
C17 Cer	$C_{35}H_{69}NO_3$	ISTD	18.20	552.535
d_{31}-C16 SM	$C_{39}H_{48}D_{31}N_2O_6P$	ISTD	14.96	734.769

ISTD: internal standard

Supplementary References

1. Rühling, M. et al. Trifunctional sphingomyelin derivatives enable nanoscale resolution of sphingomyelin turnover in physiological and infection processes via expansion microscopy. *Nature Communications* **15**, 7456 (2024).
2. Moldovan, A. et al. Chlamydia trachomatis exploits sphingolipid metabolic pathways during infection of phagocytes. *mBio*, e0398124 (2025).
3. Wagner, F. et al. Monitoring Sphingomyelin Biosynthesis at Nanoscale Resolution by Expansion Microscopy. *bioRxiv*, 2026.2005.2027.726241 (2026).
4. Lee, J.K. et al. Replication-dependent size reduction precedes differentiation in Chlamydia trachomatis. *Nature Communications* **9**, 45 (2018).
5. Rother, M., Teixeira da Costa Ana, R., Zietlow, R., Meyer Thomas, F. & Rudel, T. Modulation of Host Cell Metabolism by Chlamydia trachomatis. *Microbiology Spectrum* **7**, 10.1128/microbiolspec.bai-0012-2019 (2019).
6. Xiang, H. et al. Physiological functions and therapeutic applications of neutral sphingomyelinase and acid sphingomyelinase. *Biomedicine & Pharmacotherapy* **139**, 111610 (2021).
7. Breiden, B. & Sandhoff, K. Acid Sphingomyelinase, a Lysosomal and Secretory Phospholipase C, Is Key for Cellular Phospholipid Catabolism. *Int J Mol Sci* **22** (2021).
8. Derré, I., Swiss, R. & Agaisse, H. The Lipid Transfer Protein CERT Interacts with the Chlamydia Inclusion Protein IncD and Participates to ER-Chlamydia Inclusion Membrane Contact Sites. *PLOS Pathogens* **7**, e1002092 (2011).
9. Koch-Edelmann, S. et al. The cellular ceramide transport protein CERT promotes Chlamydia psittaci infection and controls bacterial sphingolipid uptake. *Cellular Microbiology* **19**, e12752 (2017).
