# Supplementary material for: Sphingolipids associate with the chlamydial nucleoid and mark developmental transitions in Chlamydia trachomatis
Source: Nat Commun. 2026 Sep 30;17:10367. doi: 10.1038/s41467-026-77974-3 (PMC13627705; doi:10.1038/s41467-026-77974-3)
Supplement: Supplementary file 2 [file 41467_2026_77974_MOESM2_ESM.pdf]

Description of Additional Supplementary Files:

Supplementary Movie 1: Z-stack of HeLa cells infected with *C. trachomatis* 1 h p.i. and visualized by 8x ExM. HeLa 229 cells were preloaded with TFSM2, infected with *C. trachomatis* in presence of TFSM2, and fixed 1 h p.i.. Samples were stained with BOIDPY-FL-DBCO as well as AZDye546-azide and 8-fold expanded. Z stacks of BODIPY-FL (green) and FRET (red) channels were recorded on a Leica Stellaris 5 microscope. Scale bar: 16 µm (2 µm with 8x expansion factor).

Supplementary Movie 2: Cryo-tomogram followed by a segmentation of a cryo-FIB milled EB particle. Green, membrane stack; blue, nucleoid; red, T3SSs; black, outer membrane, dark grey; inner membrane; light grey, cytoplasm. Scale bar: 200 nm
