# Supplementary material for: Sphingolipids associate with the chlamydial nucleoid and mark developmental transitions in Chlamydia trachomatis
Source: Nat Commun. 2026 Sep 30;17:10367. doi: 10.1038/s41467-026-77974-3 (PMC13627705; doi:10.1038/s41467-026-77974-3)
Supplement: Supplementary file 3 [file 41467_2026_77974_MOESM5_ESM.pdf]

nature portfolio

Corresponding author(s): Thomas Rudel

Last updated by author(s): Jul 25, 2026

Reporting Summary

Nature Portfolio wishes to improve the reproducibility of the work that we publish. This form provides structure for consistency and transparency in reporting. For further information on Nature Portfolio policies, see our Editorial Policies and the Editorial Policy Checklist.

Statistics

For all statistical analyses, confirm that the following items are present in the figure legend, table legend, main text, or Methods section.

n/a	Confirmed	
☐	☒	The exact sample size (*n*) for each experimental group/condition, given as a discrete number and unit of measurement
☒	☐	A statement on whether measurements were taken from distinct samples or whether the same sample was measured repeatedly
☐	☒	The statistical test(s) used AND whether they are one- or two-sided<br>*Only common tests should be described solely by name; describe more complex techniques in the Methods section.*
☒	☐	A description of all covariates tested
☐	☒	A description of any assumptions or corrections, such as tests of normality and adjustment for multiple comparisons
☐	☒	A full description of the statistical parameters including central tendency (e.g. means) or other basic estimates (e.g. regression coefficient) AND variation (e.g. standard deviation) or associated estimates of uncertainty (e.g. confidence intervals)
☐	☒	For null hypothesis testing, the test statistic (e.g. *F*, *t*, *r*) with confidence intervals, effect sizes, degrees of freedom and *P* value noted<br>*Give P values as exact values whenever suitable.*
☒	☐	For Bayesian analysis, information on the choice of priors and Markov chain Monte Carlo settings
☒	☐	For hierarchical and complex designs, identification of the appropriate level for tests and full reporting of outcomes
☒	☐	Estimates of effect sizes (e.g. Cohen's *d*, Pearson's *r*), indicating how they were calculated

Our web collection on statistics for biologists contains articles on many of the points above.

Software and code

Policy information about availability of computer code

Data collection	StepOne™ Software v2.3, Leica LAS AF Version 2.7.3.9723, Leica LAS X Version 4.9.0.30221, SmartSEM, SmartFIB, SerialEM v4.2, PACEtomo, SPACEtomo, MassHunter Workstation LC/MS Data Acquisition (version 10.1, Agilent Technologies), Amersham Typhoon RGB (Scansoftware Version 2.0.0.6)
Data analysis	Fiji, (Schindelin et. al 2012, doi: 10.1038/nmeth.2019, version 2.9.0), Excel (Microsoft Office, Version 2605 Build 16.0.20026.20166), GraphPad Prism Version 10.1.2 and Version 11.0.1, Design & Analysis Software Version 2.7.0. (Thermo Fisher Scientific), IMOD v5.1.0, DeepDeWedge, Dynamo v1.1.558, Dragonfly v2024.1, ChimeraX v1.9 (Plugin ArtiaX 0.6.0), Blender v4.5, MassHunter Quantitative Analysis (version 10.1, Agilent Technologies), MassHunter Qualitative Analysis (version 10.0, Agilent Technologies), R studio (R version 4.3.2)

For manuscripts utilizing custom algorithms or software that are central to the research but not yet described in published literature, software must be made available to editors and reviewers. We strongly encourage code deposition in a community repository (e.g. GitHub). See the Nature Portfolio guidelines for submitting code & software for further information.

Data

Policy information about availability of data

All manuscripts must include a data availability statement. This statement should provide the following information, where applicable:

- Accession codes, unique identifiers, or web links for publicly available datasets
- A description of any restrictions on data availability
- For clinical datasets or third party data, please ensure that the statement adheres to our policy

The cryo-electron tomograms shown in figures were uploaded to the Electron Microscopy Data Bank (EMDB) with accession numbers EMD-59116 – EMD-59119. Otherwise, all data generated or analysed during this study are included in the published article and its supplementary information files. The mass spectrometry data generated in this study are provided in the Supplementary Information and the Source Data. Source data are provided in this paper.

Research involving human participants, their data, or biological material

Policy information about studies with human participants or human data. See also policy information about sex, gender (identity/presentation), and sexual orientation and race, ethnicity and racism.

Reporting on sex and gender	n/a
Reporting on race, ethnicity, or other socially relevant groupings	n/a
Population characteristics	n/a
Recruitment	n/a
Ethics oversight	n/a

Note that full information on the approval of the study protocol must also be provided in the manuscript.

Field-specific reporting

Please select the one below that is the best fit for your research. If you are not sure, read the appropriate sections before making your selection.

[x] Life sciences [ ] Behavioural & social sciences [ ] Ecological, evolutionary & environmental sciences

For a reference copy of the document with all sections, see nature.com/documents/nr-reporting-summary-flat.pdf

Life sciences study design

All studies must disclose on these points even when the disclosure is negative.

Sample size	Samples size was determined empirically (e.g. based on previous studies Götz & Kunz et. al, https://doi.org/10.1038/s41467-020-19897-1 or Kunz &Rühling et. al https://doi.org/10.3389/fcimb.2021.644750 or Rühling&Kersting et al .https://doi.org/10.1038/s41467-024-51874-w)
Data exclusions	No data points were excluded from the analysis
Replication	Cell lines separtly seeded between replicates, replicates were performed on different days, partially experiments were preformed by different researchers. For number of independent experiments see figure legends
Randomization	The study was restricted to cell lines. Each well on the well plate was considered as independent but identical experimental unit. Thus, group allocation is irrelevant to our study.
Blinding	The investigators were not blinded to allocation during experiments and outcome assessment. Blinding was not relevant since usually structures within the same samples/image were compared (e.g. FRET efficiency inclusions vs. host cell, DNA-TFSM association or RBs vs. EBs within the same inclusion). Furthermore, analysis of these structures was often automatized e.g. setting thresholds and using the particle analyzer of Fiji.

Reporting for specific materials, systems and methods

We require information from authors about some types of materials, experimental systems and methods used in many studies. Here, indicate whether each material, system or method listed is relevant to your study. If you are not sure if a list item applies to your research, read the appropriate section before selecting a response.

Materials & experimental systems

n/a	Involved in the study
☐	☒ Antibodies
☐	☒ Eukaryotic cell lines
☒	☐ Palaeontology and archaeology
☒	☐ Animals and other organisms
☒	☐ Clinical data
☒	☐ Dual use research of concern
☒	☐ Plants

Methods

n/a	Involved in the study
☒	☐ ChIP-seq
☒	☐ Flow cytometry
☒	☐ MRI-based neuroimaging

Antibodies

Antibodies used

anti-CERT (Abcam, Cat. No. ab72536, Lot. GR251060-37, dilution 1:200), anti-chlamydial HSP60 antibody (SantaCruz, Cat. No. sc-57840, Lot. #K1220, dilution 1:400), goat anti-mouse Alexa Fluor 488 (ThermoFisher, Cat. No. A11001, dilution 1:200) , goat anti-rabbit CF568 (Sigma, Cat. No. SAB4600310, dilution 1:200)

Validation

CERT: Rabbit Polyclonal CERT antibody. Suitable for IP, WB and reacts with Human samples. Immunogen corresponding to Synthetic Peptide within Human CERT1 aa 300-350. More information can be found online. https://www.abcam.com/en-us/products/primary-antibodies/cert-antibody-ab72536
chlamydial HSP60: raised against recombinant serovar A HSP 60 of Chlamydia trachomatis origin, recommended for detection of a Cterminal region corresponding to amino acids 401-544 of HSP 60 of C. trachomatis origin by WB, IP and IF; also recommended for detection of HSP 60 of C. psittaci, N. gonorrhoeae, with minimal cross-reactivity to B. Burgdorferi. More information can be found online: https://www.scbt.com/de/p/hsp-60-antibody-a57-b9

Eukaryotic cell lines

Policy information about cell lines and Sex and Gender in Research

Cell line source(s)

HeLa cells (ATCC CCL-2), HeLa 229 cells (ATCC CCL-2.1), HeLa CERT K.O.s and parental cells were provided by Dagmar Heuer, Robert Koch-Institut, Berlin. HeLa ASM K.O.s and parental cells were provided by Martin Fraunholz, Julius-Maximilians University of Würzburg.

Authentication

None of the cell lines were authenticated

Mycoplasma contamination

Cell lines regularly are tested negative for Mycoplasma contamination by PCR

Commonly misidentified lines
(See ICLAC register)

Nocommonly misidentified cell lines were used in the study.

Plants

Seed stocks

n/a

Novel plant genotypes

n/a

Authentication

n/a
