# Supplementary material for: Sphingolipids associate with the chlamydial nucleoid and mark developmental transitions in Chlamydia trachomatis
Source: Nat Commun. 2026 Sep 30;17:10367. doi: 10.1038/s41467-026-77974-3 (PMC13627705; doi:10.1038/s41467-026-77974-3)
Supplement: Supplementary file 4 [file 41467_2026_77974_MOESM6_ESM.pdf]

nature portfolio

Peer Review File

Sphingolipids associate with the chlamydial nucleoid and mark developmental transitions in *Chlamydia trachomatis*

Corresponding Author: Professor Thomas Rudel

This file contains all reviewer reports in order by version, followed by all author rebuttals in order by version.

Version 0:

Reviewer comments:

Reviewer #1

(Remarks to the Author)
Comments to Nat Commun (NCOMMS-26-015470-T)

General Comment
This manuscript suggests that sphingomyelin (SM) regulates the genomic DNA condensation states relevant to EB-to-RB phase transitions in the obligate intracellular bacterium Chlamydia. The TFSM1/TFSM2 pair, which enables intramolecular FRET via a click chemistry reaction, is an intriguing tool for lipid cell biology. To my knowledge, the proposed concept—that sphingolipids control genome condensate states—is novel not only for Chlamydia spp. but also for other organisms. However, this intriguing argument is drawn from data using unnatural short-chain SM, raising concerns as to whether the proposed model reflects the roles of natural sphingolipids. I also identified a few other flaws. Thus, I request that the authors address these concerns. More specific comments are listed below.

Major Comments
1. Are natural SM species with long acyl chains enriched in the nucleoid of EBs compared to other membrane regions or the whole body of the EB?

2. As the N-acyl chain lengths of TFSM1/TFSM2 are much shorter than those of natural SM, the physicochemical nature of these artificial lipids differs considerably from natural SM. Most critically, rapid and spontaneous inter-membrane transfer occurs for short-chain sphingolipid analogs but not for natural SM and ceramide (e.g., PMID: 2354161; 8241188; 9220970). Why did the authors not employ artificial SM analogs with long (e.g., C16:0) N-acyl chains?

3. L300-302 and Fig. 6: The authors conclude there is an "uptake of host cell SM." This argument contradicts previous studies showing that host SMS1/SMS2 are dispensable for the infection and propagation of C. trachomatis (PMID: 31596951; 36499025). Additionally, several studies have shown that host cell CERT is important (though not essential) for Chlamydia infection/propagation (PMID: 21909260; 21731489; 8544656; 31596951; 36499025), while the authors state, "our data did not support a role for these host factors (i.e., CERT and SMases) in the enrichment of metabolized TFSM" (lines 237-238). The authors should provide compelling evidence for the uptake of "natural SM" by inclusions.
The reviewer considers an alternative possibility: long-chain natural ceramide is transferred from the host endoplasmic reticulum (ER) to inclusions, as described in previous studies, and that ceramide is then converted to SM within inclusions by host-SMS2 (PMID: 21909260) or by a putative Chlamydia-derived SMS (PMID: 31596951; 36499025). If this is the case, not only de novo synthesized ceramide but also external natural SM-derived ceramide (produced via host cell SMases) may be transferred from host cell organelles to the inclusion in a CERT-dependent manner, acting as a direct precursor of natural SM generated in inclusions. The authors should at least rule out this possibility. Otherwise, this alternative model should be included in the main conclusions and models.

4. Supp Note 5: The authors state, “We did neither detect increased expression levels of these enzymes (Supp. Fig. 6, a and b), nor did we measure an increased cellular nSMase (Supp. Fig. 6, c) or aSMase (Supp. Fig. 6, d) activity upon infection, suggesting that host cell SMases are not responsible for the enrichment of metabolized TFSMs in chlamydial inclusions.”
I disagree with this reasoning. The observation that total enzyme levels or global activities do not increase upon infection does not necessarily mean these enzymes are not involved in the process. To support such a claim, the authors should demonstrate that the genetic disruption of these enzymes or treatment with specific inhibitors does not affect TFSM behavior.

Furthermore, please refer to Major Comment #3 regarding an alternative interpretation of the CERT-independent behavior observed for short-chain TFSMs.

Minor Comments
5. L83, L87, and L92: What do the terms 4xExM, 8xExM, and pan-ExM refer to? If they are abbreviations, they should be spelled out or defined at their first appearance. I recommend briefly explaining "Expansion Microscopy" in the first paragraph of the Results section.

6. L290 and Fig 5e, f: The contents of TFSM1-ceramide and TFSM1-SM were normalized to C16:0-ceramide and C16:0-SM, respectively. Do the contents (or MS peak areas) of C16:0-ceramide and C16:0-SM per host cell number (or per protein level) change depending on Chlamydia infection or the time post-infection?

7. Tachida et al, showed that the double-disruption of SMS1 and SMS2 genes in host HeLa cells did not compromise Chlamydia infection/propagation (PMID: 31596951). Although their results are clear, they used a HeLa cell line that may differ from HeLa 229, which is widely used in Chlamydia studies. If possible, I recommend the authors verify whether SMS1/SMS2 are also dispensable for Chlamydia infection/propagation in the HeLa 229 cell line. This result may influence the authors' response to Major Comment #3.

Reviewer #2

(Remarks to the Author)
This study focuses on the role of sphingolipids in regulating the Chlamydia trachomatis developmental cycle. Sphingomyelin (SM) is known to accumulate in the cell envelope of Chlamydia. Here, the authors aim to characterize sphingolipid dynamics during the transition from the infectious elementary body (EB), a small bacterial form with condensed DNA capable of infecting cells, to the reticulate body (RB), a larger form with decondensed DNA that can replicate, and vice versa, using state-of-the-art expansion microscopy and lipid probes (e.g., trifunctional sphingomyelin, TFSM).

The authors report the novel and interesting observation that TFSM accumulates at one pole of the EB, where it co-localizes with the EB nucleoid. In the context of entry, the nucleoid/TFSM punctum appears to localize opposite to the type III secretion system that contacts the plasma membrane. This punctate colocalization of TFSM and the nucleoid is lost as EBs differentiate into RBs, concomitant with an increase in bacterial size and DNA decompaction. The authors further show that as RBs transition back to EBs, TFSM re-accumulates into puncta and loses FRET capability, consistent with its metabolism into other sphingolipid species. Using a mass spectrometry approach on whole infected cells, the authors detect metabolized ceramide and sphingomyelin species and propose that these lipids correspond to metabolized TFSM accumulating in bacteria during the transition to EBs.

The study provides novel observations on the sphingolipid composition of EBs and RBs and suggests a potential role for sphingolipid species in DNA compaction during the RB-to-EB transition. Overall, it sheds new light on the transition between these two bacterial forms, a defining characteristic of Chlamydiaceae that is critical for infection. The technical innovation is also notable, as the combination of TFSM labeling with expansion microscopy enables lipid tracking at a resolution not previously achieved. Despite its many strengths, including thorough quantification of the data, the study remains largely descriptive, and in several instances the conclusions appear somewhat overreaching, which detracts from what is otherwise a technical tour de force. The manuscript is also difficult to read, which may be in part due to the journal’s recommendations. Overall, the potential for artefacts and limitations in data interpretation dampens the enthusiasm for what may be a very impactful contribution to the field. I have listed some comments below, not in any particular order, for the authors’ consideration.

Specific comments:
- A better description of the figures in the main text, including supplementary figures, is needed. The authors should more clearly explain why each experiment was performed, what was observed (with a description of each panel rather than a single sentence), and what conclusions can reasonably be drawn, without overinterpretation. As written, large parts of the manuscript read almost like a brief report, whereas additional detail would benefit the reader. I acknowledge that the “Notes” partially address these concerns, but reformatting the manuscript would greatly benefit the reader and the review process.
- Figure 1: A clearer explanation of the differences between TFSM1 and TFSM2 is needed, as well as justification for why one is used in certain experiments versus the other.
- Figure 2: The cryo-EM data clearly show a membrane stack between the cell envelope and the nucleoid, an area that appears to correspond to the TFSM puncta observed by light microscopy. Have the authors considered that the TFSM puncta may correspond to this membrane stack rather than the nucleoid? If so, this would alter the interpretation of the observations.
- Lines 143–147: It is unclear why this experiment was performed. What specific question is being addressed, and what conclusion can be drawn?
- Line 151: How do the authors rule out that the observation is not an artifact resulting from the addition of TFSM and/or from expansion microscopy? Some controls are included, but since the study is largely observational, additional reassurance that the observations are not due to technical artefacts, potentially using complementary approaches, would be important.
- Examples of potentially overreaching conclusions include lines 172–173, 194–195, and 266–267.
- Lines 169–170 (and elsewhere): The phrasing “single accumulation” and “multiple accumulation” when referring to bacterial DNA is unclear and somewhat awkward.

• Lines 177–179: Do the authors suggest that entering EBs accumulate host lipids and incorporate them into the nucleoid prior to nucleoid decondensation? This seems somewhat inconsistent with the final model, which suggests incorporation during EB formation. Please clarify.
• Lines 194–195: Could the decrease in signal intensity be due to dilution of the signal as the bacteria increase in size?
• Lines 200–201: The conclusion is vague; please clarify what is meant by “several intermediate stages.”
• Lines 224–226: RB-to-EB differentiation is asynchronous. Do the authors suggest that a decrease in signal due to a small fraction of RBs differentiating into EBs at 24 h post-infection can be detected at the inclusion level?
• Fig. 4d–e (CtrR3 strain) appears to add limited value, as it does not substantially strengthen the model. The results are somewhat expected, as this comparison is similar to comparing wild-type inclusions at 24 h (mostly RBs) versus 48 h (mostly EBs). Additionally, the enlarged RB phenotype of the CtrR3 strain suggests defects beyond RB–EB differentiation, which may introduce additional variables and complicate interpretation.
• Figure 5 and the TFSM FRET methodology in RB versus EB: Can the authors rule out that the decrease in FRET signal (and inferred increase in metabolized TFSM) is due to (1) EBs being smaller and therefore containing less lipid available for FRET, or (2) increased lipid compaction and/or cell wall modifications in EBs affecting FRET efficiency?
• Figure 5c: What are the black dots? It is also unclear what is meant by “inclusion.”
• Figures 5c–d: The y-axis is labeled “SM content,” whereas FRET signal appears to be measured.
• Figures 5e–f and lines 362–363: The interpretation should consider that whole infected cells were analyzed, resulting in a loss of spatial resolution. Therefore, it cannot be excluded that the detected metabolized TFSM species are from the host. This point is particularly important, as the proposed model implies that bacteria uptake sphingomyelin, convert it to ceramide, and then back to sphingomyelin, which would be a complex and potentially energetically costly process. Additionally, lack of detection of other lipid species does not necessarily mean they are not produced.
• Figure 5 legend: Difficult to follow; it may help to separate panel (c) from panels (a–b).
• Lines 326–327: Do the authors suggest that chlamydial histone-like proteins do not contribute to DNA condensation?
• Lines 365–366: It is unclear what is meant by “However, the use…measured in the present study”; this sentence should be clarified.
• Lines 372–373: The data are largely observational, and there is no direct evidence for a functional role. This statement should be toned down
• Supplementary Figure 6: The legend is difficult to follow and would benefit from restructuring to describe each panel individually. In panel f, it is unclear what “2000” refers to; please clarify.

Version 1:

Reviewer comments:

Reviewer #1

(Remarks to the Author)
General Comment
I have carefully examined the revised manuscript and found that, although most of my major and minor comments were adequately addressed, a few of the most critical concerns remain unresolved. In particular, it is still unclear whether the proposed model accurately reflects the intracellular behavior and functions of natural sphingomyelin (SM). I appreciate that there are technical limitations that may have prevented the authors from fully addressing these concerns. Nevertheless, because I believe this study has the potential to make a significant scientific contribution, I encourage the authors to further improve the manuscript by addressing the remaining issues outlined below.

Major Comments
1. L190–200: pGk12a, a palmitoylated peptide carrying positive charges, is a membrane probe that presumably interacts with acidic phospholipids such as phosphatidylserine and phosphoinositides. Therefore, the new data obtained using pGk12a do not provide evidence for the enriched distribution of natural SM in the EB nucleoid. Instead, the authors should have employed SM-specific probes, such as equinatoxin II-derived probes (e.g., PMID: 41267463; 40101206), in a post-expansion staining protocol. Throughout the manuscript, the authors should carefully avoid implying that the behavior of the unnatural SM analogs reflects that of natural SM unless direct and compelling evidence for the enriched distribution of natural SM is provided.
2. L273–274: “BODIPY-FL-C12-SM, a lipid analogue that unlike TFSMs possess a naturally, even though rarely occurring, acyl chain length and hence, should adequately mimic the physiological lipid transport.” I do not agree with this argument. Although BODIPY-FL-C12-SM may be more hydrophobic than the TFSM analogs, it is still considerably less hydrophobic than C16-SM, a representative natural SM species.
3. L369–371: " These observations support a model in which TFSM is transiently metabolized to ceramide, that subsequently serves as a substrate for TFSM1-SM synthesis, resulting in the accumulation of TFSM1-SM in the inclusion." I agree with this interpretation. Although the FRET-based data presented in Fig. 5 and Supplementary Fig. 6 strongly suggest that TFSM is metabolized in infected host cells, the finding that the FRET changes are independent of host SMases or CERT does not, by itself, provide compelling evidence that the conversion of TFSMs to their corresponding ceramide analogs is independent of these host factors. Therefore, the authors should clearly state whether the conversion of TFSMs to their ceramide analogs is independent of host SMases (acid SMase and neutral SMases). If so, the authors should briefly discuss the possibility that TFSMs are metabolized by chlamydial enzymes. Conversely, if the conversion is dependent on host SMases, the authors should discuss the possibility that host SMase(s), together with CERT, contribute to the delivery of natural ceramides to the inclusion for endogenous SM synthesis. In the latter case, Fig. 7 should also be revised to reflect this possibility.

Minor Comments
4. L93: "sphingomyelin" can be replaced with the abbreviation "SM."
5. L93: "SM distribution" should be revised to, for example, "distribution of the SM derivatives" (see also Major Comment 1).
6. L109–110: "...detected a similarly localized accumulation to that observed in TFSM2-labeled samples (Fig. 1d), indicating that one pole of the EB is enriched in both sphingolipids and proteins" should be toned down. For example, replacing "indicating" with "suggesting" would be more appropriate (see also Major Comment 1).
7. L153: "globe-shaped TFSM-enriched structure": If the authors are referring to the blue-colored nucleoid region in Fig. 3b, it appears more dumpling-shaped or rugby ball-shaped (at least to me) rather than globe-shaped.

Reviewer #2

(Remarks to the Author)
Thank you for taking the time to address my comments. I don't have any further concerns.

Open Access This Peer Review File is licensed under a Creative Commons Attribution 4.0 International License, which permits use, sharing, adaptation, distribution and reproduction in any medium or format, as long as you give appropriate credit to the original author(s) and the source, provide a link to the Creative Commons license, and indicate if changes were made.
In cases where reviewers are anonymous, credit should be given to 'Anonymous Referee' and the source.
The images or other third party material in this Peer Review File are included in the article's Creative Commons license, unless indicated otherwise in a credit line to the material. If material is not included in the article's Creative Commons license and your intended use is not permitted by statutory regulation or exceeds the permitted use, you will need to obtain permission directly from the copyright holder.
To view a copy of this license, visit https://creativecommons.org/licenses/by/4.0/

REVIEWER COMMENTS

Reviewer #1 (Remarks to the Author):

Comments to Nat Commun (NCOMMS-26-015470-T)

General Comment

This manuscript suggests that sphingomyelin (SM) regulates the genomic DNA condensation states relevant to EB-to-RB phase transitions in the obligate intracellular bacterium Chlamydia. The TFSM1/TFSM2 pair, which enables intramolecular FRET via a click chemistry reaction, is an intriguing tool for lipid cell biology. To my knowledge, the proposed concept—that sphingolipids control genome condensate states—is novel not only for Chlamydia spp. but also for other organisms. However, this intriguing argument is drawn from data using unnatural short-chain SM, raising concerns as to whether the proposed model reflects the roles of natural sphingolipids. I also identified a few other flaws. Thus, I request that the authors address these concerns. More specific comments are listed below.

Major Comments

1. Are natural SM species with long acyl chains enriched in the nucleoid of EBs compared to other membrane regions or the whole body of the EB?

We share the reviewer`s interest in detecting natural sphingolipid species within the nucleoid of EBs. However, visualization of natural sphingolipids at the high resolution required for the detection of individual nucleoids cannot be achieved with currently available microscopy techniques. Recent advances in matrix-assisted laser desorption/ionization mass spectrometry imaging (MALDI-MSI) enabled the visualization of natural lipids (e.g. [1, 2]). The resolution of these highly specialized approaches are usually limited to the micrometer range (~600 nm for advanced methods [1]). Based on our ExM images, a resolution of ~50 nm is required to clearly visualized nucleoids, which is ten-fold smaller than achieved by current MALDI-MSI methods. By contrast, 8xExM on a conventional confocal microscope enables a resolution of , ExM-compatible membrane labeling was achieved recently by a molecule called pGk13a [8]. During the revision process, we stained samples with this molecule and included the resulting data in the revised manuscript (new Supp. Figure 4). Although pGk13a is not specific for sphingolipids, it enables visualization of membrane structures at ExM resolution. Similar to TFSM staining, pGk13a revealed a strong membrane staining at one EB pole that was closely associated with the nucleoid and likely corresponds to the additional membrane stacks observed in our study. Hoechst and pGk13a completely overlapped in several particles (Supp. Figure 4, c and d). However, Hoechst and pGk13a did not colocalize perfectly in all particles and often we found the two channels only partially overlapping in areas containing the nucleoid. Additionally, in pGk13a-stained samples, we did not observe the “globe-shaped protrusion” found in samples visualized by TFSM (Figure 3, e in the revised manuscript), which convinced us of the incorporation of the lipid derivative into the nucleoid. This suggests that pGk13a, unlike TFSM, does not stain the complete nucleoid and the partial signal overlap indicates a membrane-nucleoid interface. Due to the limited resolution of 8x ExM, the structure of this interface remains elusive. It is possible that the nucleoid is in close proximity to the membrane stacks or that the nucleoid partially contains membrane structure. It will be interesting to further investigate the nucleoid-membrane interface in future studies for instance by 20x ExM [9].

pGk13a was designed to label membranes. In theory, incorporation of natural lipids into the nucleoid does not necessarily need to result in formation of membranous structures, for instance when lipids form complexes with histone-like proteins or DNA. Hence, we think that these results do not necessarily contradict the incorporation of TFSM (and natural SM) into the nucleoid. However, we agree with the reviewer that although our applications are on the cutting edge of lipid visualization, we cannot unequivocally prove the incorporation of natural lipids into the chlamydial nucleoid with the data and methods currently available. We hence do no longer refer to natural lipids but “lipid derivatives” or “lipid analogs”, when we describe results obtained with TFSMs. We also discuss these limitations in the revised version of the manuscript (line 415-437 in the revised manuscript).

2. As the N-acyl chain lengths of TFSM1/TFSM2 are much shorter than those of natural SM, the physicochemical nature of these artificial lipids differs considerably from natural SM. Most critically, rapid and spontaneous inter-membrane transfer occurs for short-chain sphingolipid analogs but not for natural SM and ceramide (e.g., PMID: 2354161; 8241188; 9220970). Why did the authors not employ artificial SM analogs with long (e.g., C16:0) N-acyl chains?

We agree with the reviewer that short-chain sphingolipids have different properties compared to long-chain lipids, although, to our knowledge, none of the studies cited above has directly compared the behavior of short-chain and long-chain SM species under comparable experimental conditions.

Indeed, we synthesized and tested a α-NH_2-ω-N_3-C_{16}-ceramide. However, despite using the same labeling protocol that was successful for TFSMs, we were unable to detect this molecule within cells (**Revision Figure 1**). Several factors may account for this observation, including i.) low solubility of the molecule in culture medium, ii.) slower cellular uptake kinetics due to the longer chain length or iii.) lower staining efficiency of the click dye.

Therefore, we cannot detect long-chain lipid species with our current methodology. We addressed this limitation in the revised manuscript and discussed it together with the revision made in response to the reviewer’s first comment (line 415-437 in the revised manuscript).

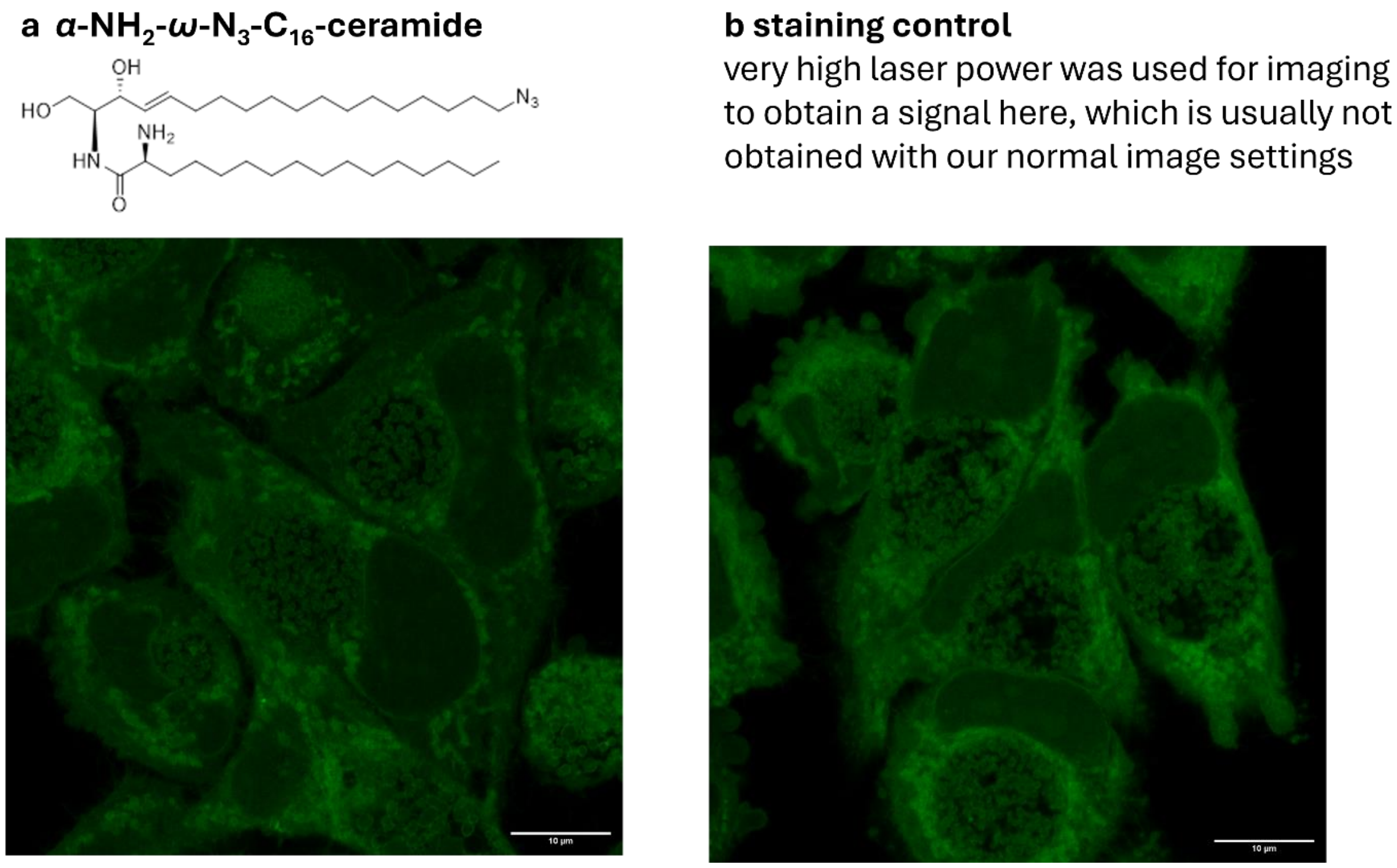

Revision Figure 1 Staining of α-NH_2-ω-N_3-C_{16}-ceramide.

HeLa 229 cells were infected in presence of 10 µM α-NH_2-ω-N_3-C_{16}-ceramide or were left untreated in case of the staining control. 24 h after infection, the cells were fixed and click chemistry was performed to stain the cells with BODIPY-FL-DBCO. Both images were acquired with the same laser settings and processed equally.

3. L300-302 and Fig. 6: The authors conclude there is an "uptake of host cell SM." This argument contradicts previous studies showing that host SMS1/SMS2 are dispensable for the infection and propagation of C. trachomatis (PMID: 31596951; 36499025). Additionally, several studies have shown that host cell CERT is important (though not essential) for Chlamydia infection/propagation (PMID: 21909260; 21731489; 8544656; 31596951; 36499025), while the authors state, "our data did not support a role for these host factors (i.e., CERT and SMases) in the enrichment of metabolized TFSM" (lines 237-238). The authors should provide compelling evidence for the uptake of "natural SM" by inclusions.

The reviewer considers an alternative possibility: long-chain natural ceramide is transferred from the host endoplasmic reticulum (ER) to inclusions, as described in previous studies, and that ceramide is then converted to SM within inclusions by host-SMS2 (PMID: 21909260) or by a putative Chlamydia-derived SMS (PMID: 31596951; 36499025). If this is the case, not only de novo synthesized ceramide but also external natural SM-derived ceramide (produced via host cell SMases) may be transferred from host cell organelles to the inclusion in a CERT-dependent manner, acting as a direct precursor of natural SM generated in inclusions. The authors should at least rule out this possibility. Otherwise, this alternative model should be included in the main conclusions and models.

We think that the uptake of non-metabolized TFSM by and its subsequent metabolization within the inclusion does not contradict previous studies suggesting that the infection is independent from SMS1/2. We do not think that SM uptake is indispensable for *C. trachomatis* infection.

In a related study [10], we investigated the metabolization of TFSM2 to TFSM2-Cer and TFSM2-SM in SMS1/2 double K.O. (dK.O.) cells by lipidomics (**Revision Figure 2**). We compared uninfected (NI) and infected (INF) samples. Importantly, the TFSM-SM metabolite was only detectable in infected SMS1/2 dK.O. cells but not in uninfected controls. This strongly suggests that resynthesis of TFSM-SM from TFSM-Cer is completely independent from the host cell SMS machinery, thereby explaining why SMS1 and 2 are dispensable for infection. Thus, our results are fully consistent with and further support the study highlighted by the reviewer.

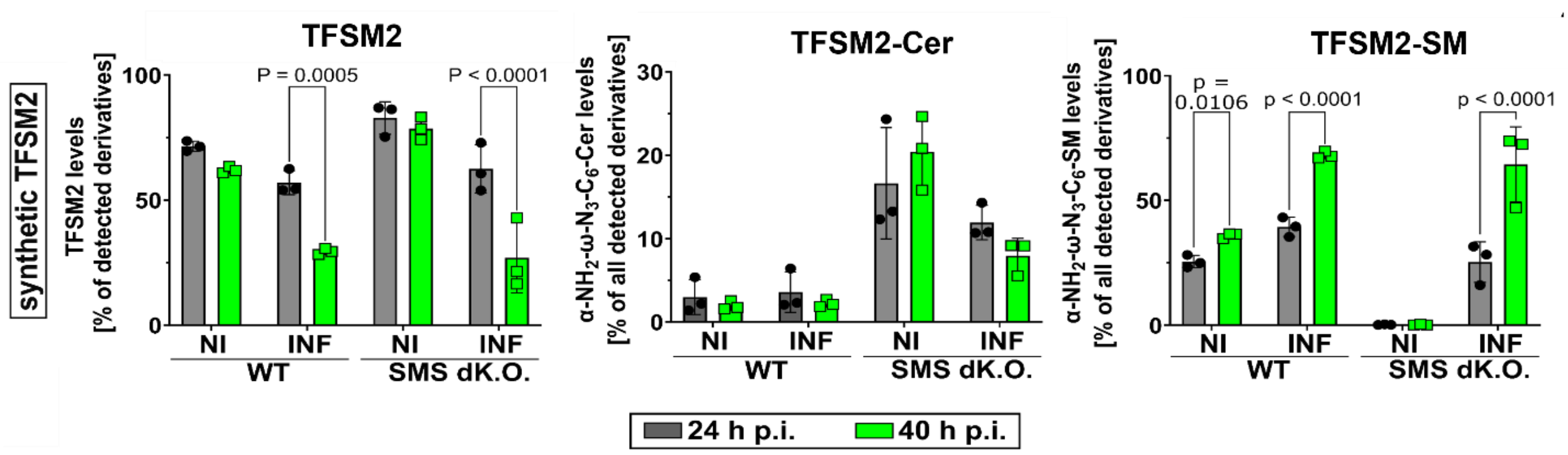

Revision Figure 2 Lipidomics analysis of TFSM2 metabolization in SM synthase 1 and 2 (SMS1/2) double K.O. (dK.O.) cells. HeLa WT or SMS dK.O. cells were infected or left uninfected in presence of 10 µM TFSM2. After the indicated time, lipids were extracted and TFSM2, TFSM2-Cer and TFSM2-SM were quantified by high pressure lipid chromatography-tandem mass spectrometry. The amount of each individual artificial lipid metabolite is expressed as proportion of all detected artificial lipid metabolites (sum of TFSM2-Cer, TFSM2 and TFSM2-SM). Modified from [10].

SM is an abundant membrane component, especially enriched in the Golgi and the plasma membrane. It is thought that *C. trachomatis* redirects Golgi-derived vesicles to the inclusion membrane (e.g.

[11],[12]) and hence, we think it is very likely that host SM is transported to the inclusion. Accordingly, several studies [13-15] suggested the transport of host-derived SM to inclusions. We performed additional experiments (see revised Supp. Figure 5, a), where we tested whether BODIPY-FL-C_{12}-SM is taken up by inclusions. This lipid analog possesses a naturally occurring even though less abundant acyl chain length. We detected uptake of BODIPY-FL-C_{12}-SM into inclusions. This was also observed in CERT K.O. cells. These findings indicate that sphingomyelin can be acquired directly from the host cell independently of CERT-mediated transport and support the conclusion that host-derived SM contributes to the sphingolipid pool within the inclusion.

In our manuscript, we describe how TFSM is metabolized within an inclusion. Our data strongly suggest that non-metabolized TFSM is taken up by the inclusion, where it is further metabolized. This was demonstrated by i.) high FRET efficiency within inclusions early during infection (16 h p.i.), which decreased at later time points (Fig. 4, a-c) and ii.) higher FRET efficiency in RBs compared to EBs (Fig. 5, a, c, d). We only detected the TFSM1-Cer and TFSM1-SM metabolites (and their enrichment during the course of in infection) by lipidomics, indicating that these are the major metabolites formed from TFSM during infection.

However, we agree with the reviewer that CERT plays a pivotal role during *C. trachomatis* infection. This was particularly demonstrated by lower infection rates in CERT-deficient cells (Supp. Figure 6, f; revised manuscript), as shown by others before (e.g. [16]). Since recruitment of CERT to inclusions was particularly strong early in infection (16 h and 24 h p.i.) and decreased thereafter (32, 40 and 46 h p.i., see Supp. Figure 7 "-AHT", revised manuscript), we think that CERT has an important function during inclusion establishment. To our knowledge, the molecular role of CERT during infection is unknown as functional analysis is lacking (e.g. investigating a CERT mutant unable to bind Cer). Consequently, it remains unclear whether infection depends on CERT-dependent Cer uptake or solely on the presence of CERT to the inclusion membrane, e.g. to establish ER-inclusion contact site as suggested by an earlier study [16].

In summary, we think that sphingolipids are acquired by inclusions via vesicular transport and CERT. Chlamydial SM synthesis might be fueled by i.) Cer acquired from the host directly or ii.) SM-derived Cer that was produced by a putative chlamydial enzyme within the inclusion. As suggested by the reviewer, we have expanded the Discussion to address the possible role of CERT in sphingolipid acquisition and metabolization during infection (see line 509-516 in the revised manuscript).

4. Supp Note 5: The authors state, "We did neither detect increased expression levels of these enzymes (Supp. Fig. 6, a and b), nor did we measure an increased cellular nSMase (Supp. Fig. 6, c) or aSMase (Supp. Fig. 6, d) activity upon infection, suggesting that host cell SMases are not responsible for the enrichment of metabolized TFSMs in chlamydial inclusions."
I disagree with this reasoning. The observation that total enzyme levels or global activities do not increase upon infection does not necessarily mean these enzymes are not involved in the process. To support such a claim, the authors should demonstrate that the genetic disruption of these enzymes or treatment with specific inhibitors does not affect TFSM behavior. Furthermore, please refer to Major Comment #3 regarding an alternative interpretation of the CERT-independent behavior observed for short-chain TFSMs.

We agree with the reviewer and therefore measured the metabolization of TFSM within inclusions in ASM K.O. cells [17] and/or in presence of the NSM inhibitor GW4869 [18]. TFSM metabolism was quantified by FRET acceptor bleaching (Supp. Figure 6, e and line 329-332 in the revised manuscript). TFSM metabolism was still observed under all conditions tested during infection. These results indicate

that host ASM and NSM activities are not required for TFSM conversion during infection, suggesting that TFSM metabolization occurs within the inclusion and may be catalyzed by a bacterial enzyme.

Minor Comments

5. L83, L87, and L92: What do the terms 4xExM, 8xExM, and pan-ExM refer to? If they are abbreviations, they should be spelled out or defined at their first appearance. I recommend briefly explaining "Expansion Microscopy" in the first paragraph of the Results section.

We added a paragraph to the introduction that explains ExM and clarifies the terminology (see line 77-85 in the revised manuscript)

6. L290 and Fig 5e, f: The contents of TFSM1-ceramide and TFSM1-SM were normalized to C16:0-ceramide and C16:0-SM, respectively. Do the contents (or MS peak areas) of C16:0-ceramide and C16:0-SM per host cell number (or per protein level) change depending on Chlamydia infection or the time post-infection?

We did not determine cell numbers or protein levels of samples analyzed by lipidomics. However, we showed previously that SM levels are constant during infection with *C. trachomatis* of macrophages and epithelial cells, while ceramide levels changed [19].

The TFSM1-Cer and TFSM1-SM content was normalized to the sum of C16:0 SM and C16:0 ceramide. Since the C16 SM content of cells is ~50-fold higher than the C16 Cer content, changes in C16 Cer are negligible.

For the revision, we used a different normalization strategy that was also used to analyze data obtained with TFSM2 (**Revision Figure 2**, [10]). We express the abundance of TFSM1, TFSM1-SM and TFSM1-Cer as proportion of the sum of these three metabolites (**Revision Figure *3***). Since we did not detect any other TFSM1-related molecules during our lipidomics screen (Figure 6, e and f, in the revised manuscript), we assume that other metabolites are formed only in negligible amounts. This normalization method is completely independent from the cell number. The results are similar to data normalized to the sum of C16:0 SM and C16:0 ceramide. TFSM1-Cer and TFSM1-SM levels increased while TFSM1 decreased compared to uninfected controls. Hence, we can exclude that changes in the abundance of TFSM1 metabolites during infection is due to normalization artifacts.

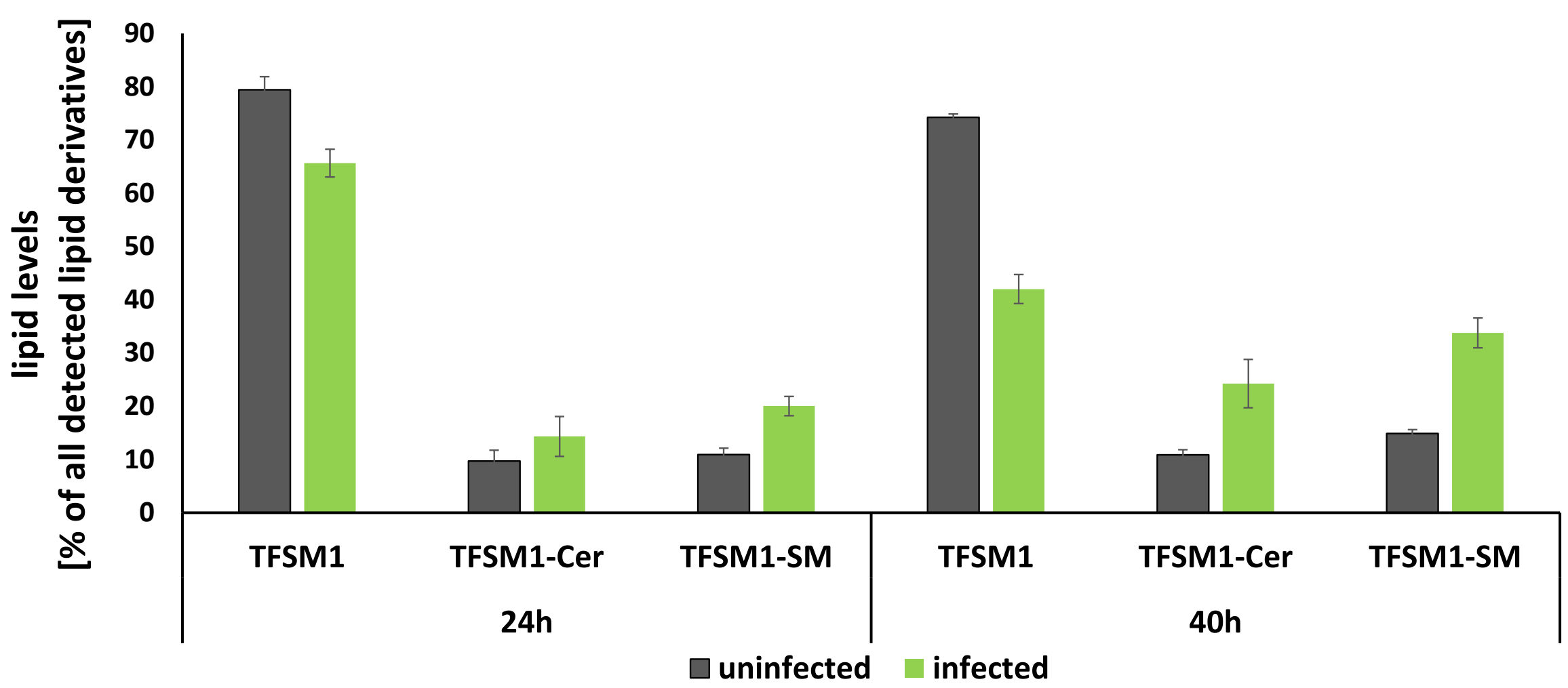

Revision Figure 3 Alternative normalization approach for lipidomics data

7. Tachida et al, showed that the double-disruption of SMS1 and SMS2 genes in host HeLa cells did not compromise Chlamydia infection/propagation (PMID: 31596951). Although their results are clear, they used a HeLa cell line that may differ from HeLa 229, which is widely used in Chlamydia studies. If possible, I recommend the authors verify whether SMS1/SMS2 are also dispensable for Chlamydia infection/propagation in the HeLa 229 cell line. This result may influence the authors' response to Major Comment #3.

We appreciate the reviewer's concern regarding the use of different HeLa cell lines. However, we so far did not observe any differences between "HeLa" and "HeLa229" regarding the metabolization of TFSM (**Revision Figure 4**). All experiments investigating host factors, including CERT [Supp Figure 6, g, f (accidentally labelled as "HeLa 2000" in first submission) and Supp. Figure 7], SMases (Supp Figure 6, a-e) or SM synthases (**Revision Figure 5**) were performed in "HeLa". Results are also in line with available literature (infection depends on CERT but not SMS1 and 2).

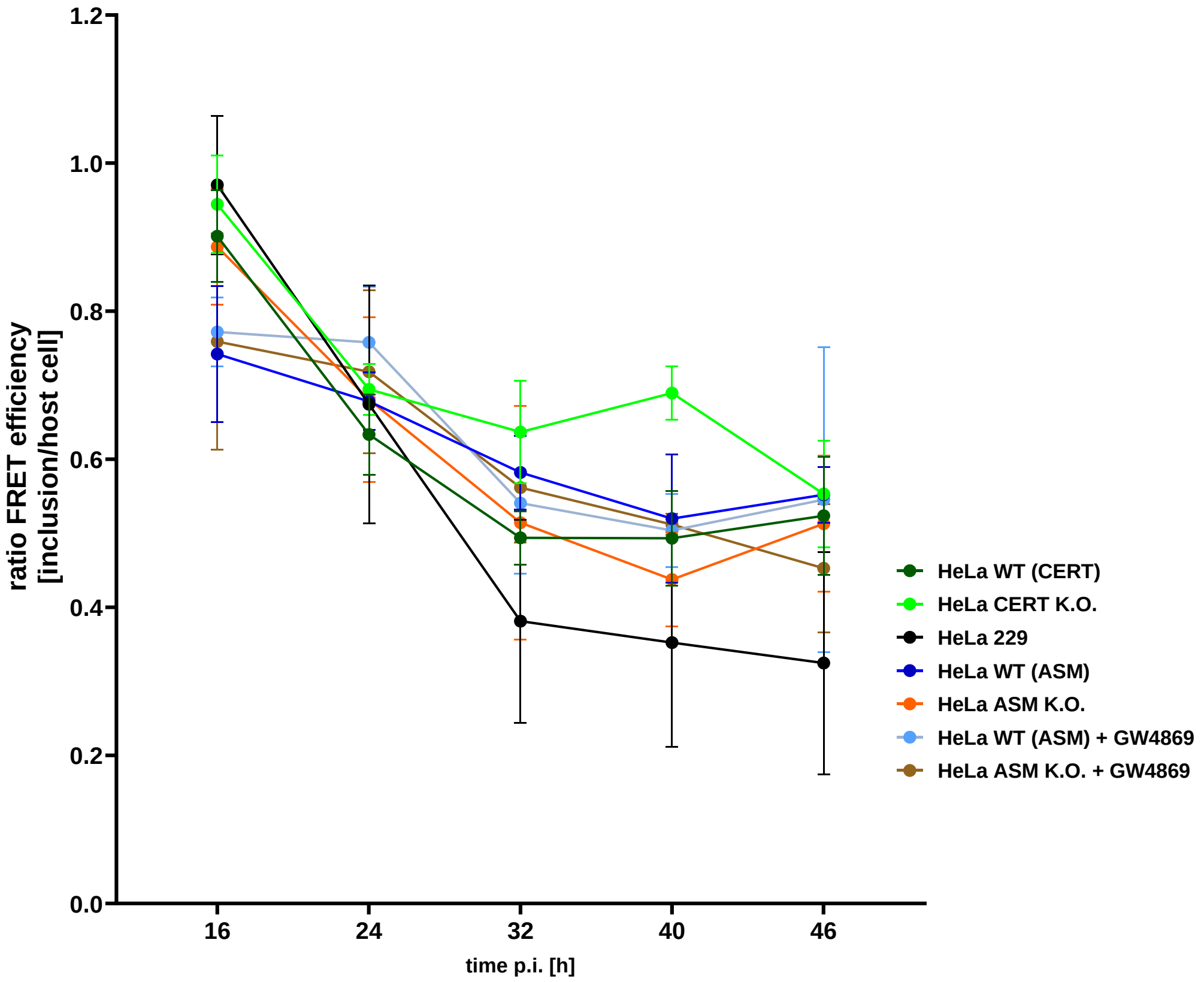

Revision Figure 4 Measurements of TFSM metabolization in *C. trachomatis* inclusions in different HeLa cell lines
The indicated cell lines were infected in presence of 10 µM TFSM2. At the indicated time point, samples were fixed stained with BODIPY-FL-DBCO and AZDye 546 azide and FRET efficiencies in inclusions and host cell areas were determined. FRET efficiencies ratios "inclusion vs. host cell" were calculated.

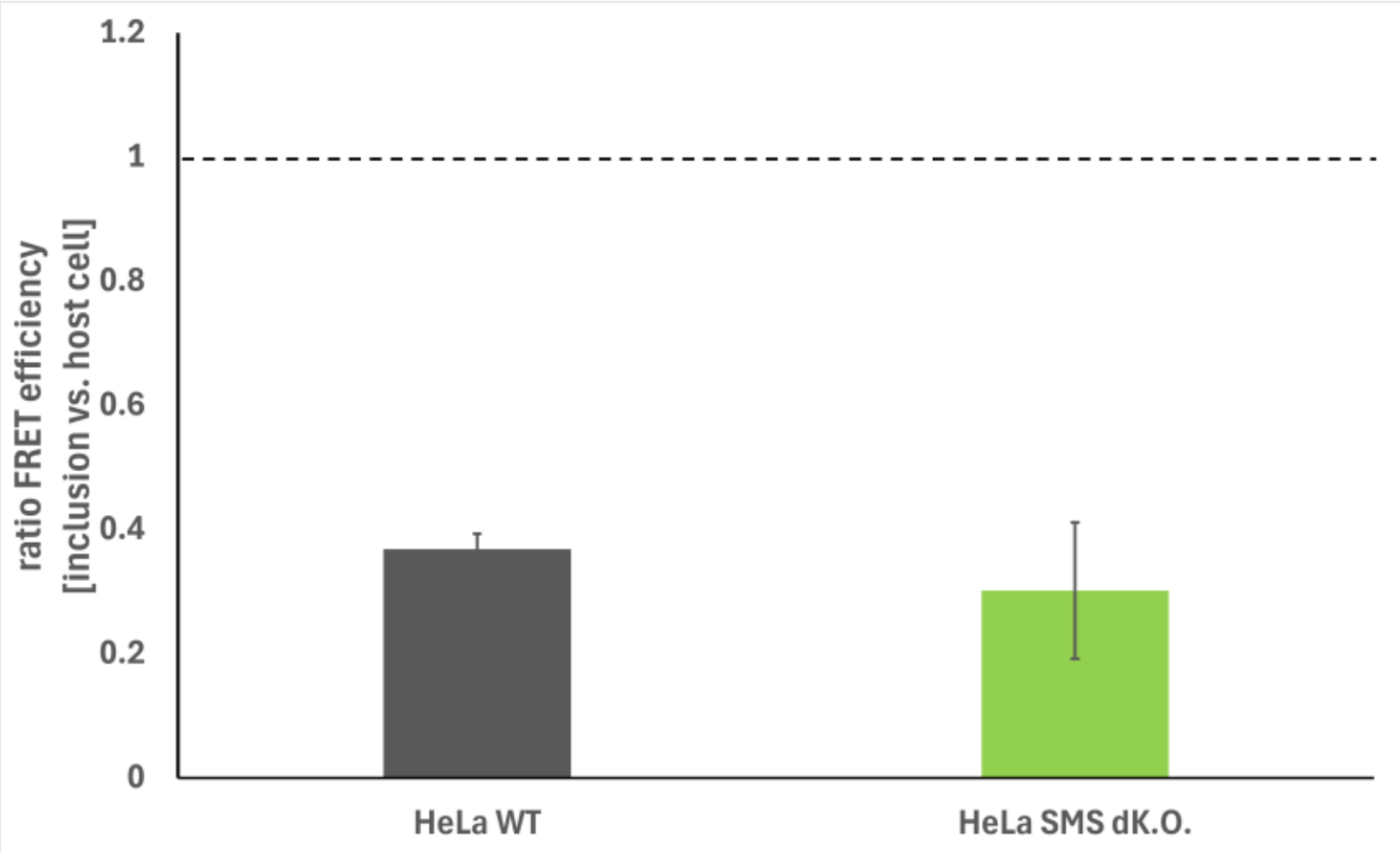

Revision Figure 5 HeLa WT or SMS dK.O. cells were infected in presence of 10 µM TFSM2.
At 32 h p.i., samples were fixed stained with BODIPY-FL-DBCO and AZDye 546 azide and FRET efficiencies in inclusions and host cell areas were determined. FRET efficiencies ratios "inclusion vs. host cell" were calculated. Dotted line indicates relative FRET efficiency of host cell areas.

Since *C. trachomatis* can synthesis SM in absence of the host cell SM synthases SMS1 and SMS2 (**Revision Figure 2** and [10]), we think that the infection in general is independent from host SMS1/2. Nevertheless, we cannot exclude the possibility that the relative contribution of individual lipid metabolic pathways may vary between cell types. Therefore, characterizing lipid metabolism during *C. trachomatis* infection in additional cellular models, including HeLa 229, will be an interesting question for future investigations.

Reviewer #2 (Remarks to the Author):

This study focuses on the role of sphingolipids in regulating the Chlamydia trachomatis developmental cycle. Sphingomyelin (SM) is known to accumulate in the cell envelope of Chlamydia. Here, the authors aim to characterize sphingolipid dynamics during the transition from the infectious elementary body (EB), a small bacterial form with condensed DNA capable of infecting cells, to the reticulate body (RB), a larger form with decondensed DNA that can replicate, and vice versa, using state-of-the-art expansion microscopy and lipid probes (e.g., trifunctional sphingomyelin, TFSM).

The authors report the novel and interesting observation that TFSM accumulates at one pole of the EB, where it co-localizes with the EB nucleoid. In the context of entry, the nucleoid/TFSM punctum appears to localize opposite to the type III secretion system that contacts the plasma membrane. This punctate colocalization of TFSM and the nucleoid is lost as EBs differentiate into RBs, concomitant with an increase in bacterial size and DNA decompaction. The authors further show that as RBs transition back to EBs, TFSM re-accumulates into puncta and loses FRET capability, consistent with its metabolism into other sphingolipid species. Using a mass spectrometry approach on whole infected cells, the authors detect metabolized ceramide and sphingomyelin species and propose that these lipids correspond to metabolized TFSM accumulating in bacteria during the transition to EBs.

The study provides novel observations on the sphingolipid composition of EBs and RBs and suggests a potential role for sphingolipid species in DNA compaction during the RB-to-EB transition. Overall, it sheds new light on the transition between these two bacterial forms, a defining characteristic of Chlamydiaceae that is critical for infection. The technical innovation is also notable, as the combination of TFSM labeling with expansion microscopy enables lipid tracking at a resolution not previously

achieved. Despite its many strengths, including thorough quantification of the data, the study remains largely descriptive, and in several instances the conclusions appear somewhat overreaching, which detracts from what is otherwise a technical tour de force. The manuscript is also difficult to read, which may be in part due to the journal's recommendations. Overall, the potential for artefacts and limitations in data interpretation dampens the enthusiasm for what may be a very impactful contribution to the field. I have listed some comments below, not in any particular order, for the authors' consideration.

Specific comments:

• A better description of the figures in the main text, including supplementary figures, is needed. The authors should more clearly explain why each experiment was performed, what was observed (with a description of each panel rather than a single sentence), and what conclusions can reasonably be drawn, without overinterpretation. As written, large parts of the manuscript read almost like a brief report, whereas additional detail would benefit the reader. I acknowledge that the "Notes" partially address these concerns, but reformatting the manuscript would greatly benefit the reader and the review process.

We substantially revised the manuscript with the goal of improving clarity and readability and hope that the revised version is now easier to follow. We also moved Supp. Figure 3 and the corresponding Supp. Note into the main text (as Figure 2 in the revised manuscript).

We also carefully reassessed our interpretations and removed or revised statements that could be viewed as overinterpretations of the data, thereby ensuring that our conclusions more closely reflect the experimental evidence.

• Figure 1: A clearer explanation of the differences between TFSM1 and TFSM2 is needed, as well as justification for why one is used in certain experiments versus the other.

In general, both molecules can be used for the same applications including microscopic visualization of cellular SM distribution by ExM, FRET measurements and lipidomics. We so far have not observed differences between the two compounds during *C. trachomatis* infection [7, 10, 19]. However, depending on the application, usage of TFSM1 might be advantageous compared to TFSM2 and vice versa. We think that the differences are minor.

We explain these differences of the two compounds more precisely in a Supp. Note (Supp. Note 1 in the revised manuscript).

• Figure 2: The cryo-EM data clearly show a membrane stack between the cell envelope and the nucleoid, an area that appears to correspond to the TFSM puncta observed by light microscopy. Have the authors considered that the TFSM puncta may correspond to this membrane stack rather than the nucleoid? If so, this would alter the interpretation of the observations.

We think that the TFSM accumulation also corresponds to the membrane stack. This stack and the lipid therein might tether the nucleoid to the inner bacterial membrane. However, the globe-shape TFSM protrusion into the EB cytosol, which strongly colocalizes with the DNA, suggests that TFSM is also located within the nucleoid.

To further investigate this possibility, we performed additional experiments during the revision using pGk13a, a recently developed ExM-compatible membrane probe [8]. While pGk13a enables high-resolution of membrane structures, it does not directly label or visualize lipids.

We observed a membrane accumulation on one EB cell pole in samples stained with pGk13a (Supp. Figure 4 in the revised manuscript), which presumably corresponds to the membrane stack/TFSM accumulation observed previously. However, we did not observe a globe-shape protrusions into the EB cytosol with pGk13a staining, even though, in some but not all particles, pGk13a strongly colocalized with Hoechst (Supp. Figure 4, c and d). Because of these differences between TFSM and pGk13a staining, we think that TFSM is distributed within the nucleoid.

The partial overlap between pGk13a and Hoechst that we observed in several particles (Supp. Figure 4, e and f) is difficult to interpret. Due to the limited resolution of 8x ExM, the structural interface between DNA and membranes is unclear. This is an important question that needs to be addressed in future studies e.g. by ExM protocols with enhanced expansion factor [9].

More generally, the role of lipids in nucleoid organization remains unresolved. It is currently unclear whether lipids constitute a structural component of the nucleoid itself or whether they primarily mediate the attachment of the nucleoid to the membrane stack. To acknowledge these limitations and discuss the possible models, we have added a paragraph to the Discussion (line 415-437 in the revised manuscript).

• Lines 143–147: It is unclear why this experiment was performed. What specific question is being addressed, and what conclusion can be drawn?

This experiment was performed to exclude staining artifacts by the click dyes. This was clarified in the revised manuscript (line 177).

• Line 151: How do the authors rule out that the observation is not an artifact resulting from the addition of TFSM and/or from expansion microscopy? Some controls are included, but since the study is largely observational, additional reassurance that the observations are not due to technical artefacts, potentially using complementary approaches, would be important.

To further exclude expansion artifacts, we assessed the expansion of RBs by measuring the particle diameter in 4x ExM vs. 8x ExM images (see Supp. Figure 2, e and f). Because RBs are less densely packed than EBs or nucleoids, they are expected to undergo complete and isotropic expansion and therefore serve as a suitable internal reference. We did not detect significant changes between the expansion of RBs, EBs or nucleoids.

In 8-fold expanded images, nucleoids/lipid accumulations had a physical size of ~0.75 µm (if not corrected for the expansion factor). This is almost as large as an RB in unexpanded samples.

There is currently no evidence suggesting anisotropic expansion of chlamydial particles. This was also demonstrated in a previous study, where we determined the distance between inner and our bacterial membrane in expanded images [6]. The distance was similar to values determined by electron microscopy. Please also refer to another study, where we used similar protocols to establish ExM of a variety of pathogens [20].

Nevertheless, we cannot fully exclude the possibility that the addition of TFSM itself influences lipid organization or distribution. In this regard, pGk13a provides an important complementary approach because it is applied after fixation and therefore should not affect membrane biology. However, as discussed in our response to a previous comment, pGk13a and TFSM staining patterns were not

identical. Consequently, while our data argue against expansion-induced artifacts, the precise extent to which TFSM faithfully reflects the distribution of endogenous sphingolipids remains a limitation of the current methodology.

Detection of natural lipids in the nucleoid by fluorescence microscopy is not possible. Natural lipids are not linked to the hydrogel unless chemically modified, and limited options for staining lipids are available. Matrix-assisted laser desorption/ionization mass spectrometry imaging (MALDI-MSI) enabled the visualization of natural lipids (e.g. [1, 2]). The resolution of these highly specialized approaches are usually limited to the micrometer range (~600 nm for advanced methods [1]). Based on our ExM images, a resolution of ~50 nm is required to clearly visualize nucleoids. This is roughly an order of magnitude higher than the spatial resolution currently achieved by MALDI-MSI approaches. By contrast, 8xExM on a conventional confocal microscope enables a resolution of

We discuss these limitations (especially the lack of evidence for natural lipid species within the nucleoid) in the revised version of the manuscript (line 415-437).

• Examples of potentially overreaching conclusions include lines 172–173, 194–195, and 266–267.

- **Line 172-173 (Line 234-235 in the revised manuscript): "During the first 6 h of infection, particle sizes increased from 200-300 nm of EBs to almost 1 µm, which is comparable to the size of RBs."**

We thank the reviewer for identifying this flaw. In the revised manuscript, we quantified the diameter of intracellular particles within the first 6 h of infection and compared them to "real" RBs 32 h p.i. (see Supp Figure 3, c, in the revised manuscript). The average particle size increased from ~200 nm at 1 h p.i. to 750 nm at 6 h p.i., while RBs at 32 h p.i. possess a size of ~1 µm. Hence, not all particles reached RB size at 6 h p.i.. We modified our conclusion accordingly (see Line 234-235 in the revised manuscript). It now reads:
"During the first 6 h of infection, particle diameters increased from ~200 nm to 750 nm, while after 32 h p.i. RBs in average measured 1 µm in size.

- **Line 194-195: "This suggested that dissociation of lipids from nucleoids requires intracellular stimuli."**

We removed this conclusion from the manuscript.

- **Line 266–267 (line 355-367 in the revised manuscript): "In summary, our data suggests that membrane-associated RBs take up native TFSM from host cells, which is metabolized after RBs detach from the inclusion membrane and differentiate into EBs (**Error! Reference source not found.**, d)."**

We changed the conclusion to: "In summary, our data suggest that membrane-associated RBs predominantly take up native TFSM, which is metabolized during RB-to-EB differentiation (**Fig. 6**, d) and subsequently accumulates on the nucleoid-containing EB cell pole (**Fig. 1**, **Fig. 2**)."

• Lines 169–170 (and elsewhere): The phrasing "single accumulation" and "multiple accumulation" when referring to bacterial DNA is unclear and somewhat awkward.

We agree with the reviewer and, in this context, replaced the word “accumulation” with “foci” in the revised manuscript.

• Lines 177–179: Do the authors suggest that entering EBs accumulate host lipids and incorporate them into the nucleoid prior to nucleoid decondensation? This seems somewhat inconsistent with the final model, which suggests incorporation during EB formation. Please clarify.

As we detected higher BODIPY-FL fluorescence intensity in nucleoids of extracellular EBs at 1 h p.i. vs. 5 min p.i. (Figure 4d), we think that host lipids are incorporated into the membranes/nucleoids of EBs attached to the plasma membrane. This lipid incorporation is not necessarily contradictive to the release of lipids from the nucleoid during EB-RB differentiation. Incorporated lipids could be metabolized e.g. by a chlamydial SMase, which could initiate the dissociation of lipids from the nucleoid. Alternatively, the lipids that are “freshly” incorporated into the nucleoid could trigger lipid release from DNA (e.g. negatively charged lipids). In this scenario, EBs could sense their lipid environment and initiate nucleoid remodeling upon encountering certain lipid species , such as negatively charged phosphoinositides that are enriched in phagosomal membranes [23-25].

The hypothesis is further clarified in the discussion (see lines 452-459 in the revised manuscript).

• Lines 194–195: Could the decrease in signal intensity be due to dilution of the signal as the bacteria increase in size?

The quantification is an average over all measured particles/nucleoids. Despite the classification between extra- and intracellular particles, we detect EBs in different developmental stages, as EBs are likely continuously taken up by the host cells during our assay. We observed EBs (or intermediate stages) with nucleoids devoid of lipids already after 1h. The size of these particles (and their nucleoids) was comparable to extracellular EBs that should not have underwent any differentiation. Hence, we think that the decrease is not due to increase in bacteria size.

• Lines 200–201: The conclusion is vague; please clarify what is meant by “several intermediate stages.”

We changed the conclusion to (line 247-250 in the revised manuscript):

“These data indicate that nucleoid decondensation during EB-to-RB differentiation is a continuous process that proceeds within the first 6 h of infection through several morphologically distinct stages. These stages can be distinguished by the degree of DNA-TFSM association and the number and distribution of DNA foci.“

• Lines 224–226: RB-to-EB differentiation is asynchronous. Do the authors suggest that a decrease in signal due to a small fraction of RBs differentiating into EBs at 24 h post-infection can be detected at the inclusion level?

The decreased FRET efficiency measured in whole inclusions at 24 h p.i., is unlikely to arise solely from the presence of EBs. Additionally, RBs that are not attached to the inclusion membrane (“other RBs”) or IBs possess lower FRET efficiencies than host cells (see Figure 6, d). This is also visualized in Figure 6,a (24h p.i.), where particles with a “green” appearance (high donor, low FRET => metabolized TFSM) can be found in the inclusion lumen. Probably, most of these particles are not EBs. Consistently, the FRET efficiency in whole inclusions drops further, when inclusions contain larger numbers of mature EBs (after 32h p.i., see Figure 5, b), suggesting that metabolized TFSM is predominantly present in these particles during/after redifferentiation.

• Fig. 4d–e (CtrR3 strain) appears to add limited value, as it does not substantially strengthen the model. The results are somewhat expected, as this comparison is similar to comparing wild-type inclusions at 24 h (mostly RBs) versus 48 h (mostly EBs). Additionally, the enlarged RB phenotype of the CtrR3 strain suggests defects beyond RB–EB differentiation, which may introduce additional variables and complicate interpretation.

We appreciate the reviewer's point that the results obtained with the CtrR3 strain are broadly consistent with the comparison between wild-type inclusions at early and late stages of infection. However, we believe that this experiment provides an important independent control for our model. In wild-type infections, developmental progression and TFSM metabolism occur simultaneously, making it difficult to distinguish whether TFSM metabolism is merely correlated with developmental stage or is linked to the RB-to-EB transition itself.

The CtrR3 strain allows us to address this question more directly because RBs are developmentally arrested and fail to efficiently differentiate into EBs. Under these conditions, TFSM metabolism was strongly reduced, despite prolonged intracellular growth. Thus, the CtrR3 experiment supports the conclusion that TFSM metabolism is associated with developmental progression rather than simply with the passage of time during infection.

We agree that the enlarged RB phenotype of the CtrR3 strain may reflect additional physiological alterations beyond the developmental block. Therefore, we do not interpret this experiment in isolation. Rather, we view it as a complementary line of evidence that is consistent with, and independently supports, the observations made in wild-type infections showing increased TFSM metabolism during RB-to-EB differentiation.

We added a paragraph to the discussion (line 512-517, revised manuscript).

• Figure 5 and the TFSM FRET methodology in RB versus EB: Can the authors rule out that the decrease in FRET signal (and inferred increase in metabolized TFSM) is due to (1) EBs being smaller and therefore containing less lipid available for FRET, or (2) increased lipid compaction and/or cell wall modifications in EBs affecting FRET efficiency?

(1) FRET efficiency measurements by acceptor bleaching is independent from local lipid concentration. In our assay, FRET efficiency reflects the ratio between non-metabolized TFSM (containing donor + acceptor) or metabolized TFSM (only containing the donor).
(2) Lipid compaction/distribution can indeed influence donor fluorescence intensity. We showed this previously (Figure 7 in [7]). Specifically, densely packed lipid assemblies, such as lipid microdomains, could bring membrane-integral donor fluorophores (BODIPY-FL/donor in our TFSM experiments) in close proximity, resulting in self-quenching and/or donor-donor interactions. However, if the reduced FRET efficiencies observed in our study were caused by lipid compaction, these effects should be abolished by ExM, which physically separates individual lipid molecules and greatly increases the distance between fluorophores. Since we observed the decrease in FRET efficiency also in ExM samples, we think that lipid compaction as cause for decreased FRET efficiencies can be excluded

• Figure 5c: What are the black dots? It is also unclear what is meant by “inclusion.

We added a description to the legend of this Figure (Figure 6 in the revised manuscript).

• Figures 5c–d: The y-axis is labeled “SM content,” whereas FRET signal appears to be measured.

We changed the axis titles.

• Figures 5e–f and lines 362–363: The interpretation should consider that whole infected cells were analyzed, resulting in a loss of spatial resolution. Therefore, it cannot be excluded that the detected metabolized TFSM species are from the host. This point is particularly important, as the proposed model implies that bacteria uptake sphingomyelin, convert it to ceramide, and then back to sphingomyelin, which would be a complex and potentially energetically costly process. Additionally, lack of detection of other lipid species does not necessarily mean they are not produced.

We thank the reviewer for this important comment. We agree that our lipidomics analyses were performed on whole infected cells and therefore lack the spatial resolution required to directly assign detected TFSM metabolites to either the host cell or the inclusion. We now explicitly acknowledge this limitation in the revised manuscript. Nevertheless, several independent observations suggest that TFSM metabolism occurs predominantly within the inclusion:

i.) FRET efficiency within host cell areas only slightly decreased between 16 h and 46 h p.i., while the FRET efficiency within inclusions declined dramatically over the same period. This suggests a massive enrichment of TFSM metabolites within inclusions (Fig. 5, a and b, in the revised manuscript).

ii.) Membrane-associated RBs possess FRET efficiencies similar to the host cell, while EBs showed significantly lower FRET efficiencies (Fig. 5, d). This indicates that non-metabolized TFSM is taken up into the inclusions and is metabolized during the RB-to-EB differentiation.

iii.) In a related manuscript ([10] and Revision Figure 1), we show that the complex TFSM-to-TFSM-SM conversion is independent from the host cell SM synthesis machinery. This strongly suggests that the TFSM-SM resynthesis is catalyzed by a chlamydial enzyme within the inclusion.

We altered the discussion in the revised manuscript accordingly (line 488-493, revised manuscript).

We agree with the reviewer that other TFSM metabolites might be formed during infection that, due to low abundancy, could not be detected with our lipidomics approach. We added an explanation in the revised version of the manuscript (line 484-488).

• Figure 5 legend: Difficult to follow; it may help to separate panel (c) from panels (a–b).

We changed the figure legend as suggested.

• Lines 326–327: Do the authors suggest that chlamydial histone-like proteins do not contribute to DNA condensation?

A recent preprint suggests that nucleoids are formed in absence of the histone-like protein hctA and hctB [26]. However, we have no functional evidence that lipids directly contribute to DNA condensation in *C. trachomatis*. We hence toned down our statement and added the above-mentioned study to the discussion (line 442-443, revised manuscript).

• Lines 365–366: It is unclear what is meant by “However, the use…measured in the present study”; this sentence should be clarified.

We added an explanation to the discussion (line 495-502, revised manuscript).

• Lines 372–373: The data are largely observational, and there is no direct evidence for a functional role. This statement should be toned down

We toned down our conclusion (line 517-522, revised manuscript).

• Supplementary Figure 6: The legend is difficult to follow and would benefit from restructuring to describe each panel individually. In panel f, it is unclear what "2000" refers to; please clarify.

We changed the figure legend accordingly and removed the "2000" that has been accidentally added.

References

1. Niehaus, M., et al., *Transmission-mode MALDI-2 mass spectrometry imaging of cells and tissues at subcellular resolution.* Nature Methods, 2019. **16**(9): p. 925-931.
2. Capolupo, L., et al., *Sphingolipids control dermal fibroblast heterogeneity.* Science, 2022. **376**(6590): p. eabh1623.
3. Honigmann, A., et al., *Scanning STED-FCS reveals spatiotemporal heterogeneity of lipid interaction in the plasma membrane of living cells.* Nature Communications, 2014. **5**(1): p. 5412.
4. White, B.M., et al., *Lipid Expansion Microscopy.* Journal of the American Chemical Society, 2022. **144**(40): p. 18212-18217.
5. Mori, T. and K.G.N. Suzuki, *Single-Molecule Imaging and Super-Resolution Microscopy of Lipid Domains in Cell Membranes Using Lipid-Binding Proteins and Fluorophore-Conjugated Lipid Analogs.* Membranes (Basel), 2025. **15**(10).
6. Gotz, R., et al., *Nanoscale imaging of bacterial infections by sphingolipid expansion microscopy.* Nat Commun, 2020. **11**(1): p. 6173.
7. Rühling, M., et al., *Trifunctional sphingomyelin derivatives enable nanoscale resolution of sphingomyelin turnover in physiological and infection processes via expansion microscopy.* Nature Communications, 2024. **15**(1): p. 7456.
8. Shin, T.W., et al., *Dense, continuous membrane labeling and expansion microscopy visualization of ultrastructure in tissues.* Nature Communications, 2025. **16**(1): p. 1579.
9. Wang, S., et al., *Single-shot 20-fold expansion microscopy.* Nature Methods, 2024. **21**(11): p. 2128-2134.
10. Wagner, F., et al., *Monitoring Sphingomyelin Biosynthesis at Nanoscale Resolution by Expansion Microscopy.* bioRxiv, 2026: p. 2026.05.27.726241.
11. Carabeo, R.A., D.J. Mead, and T. Hackstadt, *Golgi-dependent transport of cholesterol to the Chlamydia trachomatis inclusion.* Proceedings of the National Academy of Sciences, 2003. **100**(11): p. 6771-6776.
12. Pokrovskaya, I.D., et al., *Chlamydia trachomatis hijacks intra-Golgi COG complex-dependent vesicle trafficking pathway.* Cell Microbiol, 2012. **14**(5): p. 656-68.
13. Hackstadt, T., et al., *Chlamydia trachomatis interrupts an exocytic pathway to acquire endogenously synthesized sphingomyelin in transit from the Golgi apparatus to the plasma membrane.* Embo j, 1996. **15**(5): p. 964-77.
14. Scidmore, M.A., E.R. Fischer, and T. Hackstadt, *Sphingolipids and glycoproteins are differentially trafficked to the Chlamydia trachomatis inclusion.* J Cell Biol, 1996. **134**(2): p. 363-74.
15. Hackstadt, T., M.A. Scidmore, and D.D. Rockey, *Lipid metabolism in Chlamydia trachomatis-infected cells: directed trafficking of Golgi-derived sphingolipids to the chlamydial inclusion.* Proceedings of the National Academy of Sciences, 1995. **92**(11): p. 4877-4881.

16. Derré, I., R. Swiss, and H. Agaisse, *The Lipid Transfer Protein CERT Interacts with the Chlamydia Inclusion Protein IncD and Participates to ER-Chlamydia Inclusion Membrane Contact Sites.* PLOS Pathogens, 2011. **7**(6): p. e1002092.
17. Rühling, M., et al., *A Novel Rapid Host Cell Entry Pathway Determines Intracellular Fate of Staphylococcus aureus.* eLife, 2026.
18. Luberto, C., et al., *Inhibition of tumor necrosis factor-induced cell death in MCF7 by a novel inhibitor of neutral sphingomyelinase.* J Biol Chem, 2002. **277**(43): p. 41128-39.
19. Moldovan, A., et al., *Chlamydia trachomatis exploits sphingolipid metabolic pathways during infection of phagocytes.* mBio, 2025: p. e0398124.
20. Kunz & Rühling, M., et al., *The Expandables: Cracking the Staphylococcal Cell Wall for Expansion Microscopy.* Front Cell Infect Microbiol, 2021. **11**: p. 644750.
21. Barry, C.E., S.F. Hayes, and T. Hackstadt, *Nucleoid Condensation in <i>Escherichia coli</i> That Express a Chlamydial Histone Homolog.* Science, 1992. **256**(5055): p. 377-379.
22. Grieshaber, N.A., et al., *Chlamydial histone-DNA interactions are disrupted by a metabolite in the methylerythritol phosphate pathway of isoprenoid biosynthesis.* Proceedings of the National Academy of Sciences, 2004. **101**(19): p. 7451-7456.
23. Wallroth, A. and V. Haucke, *Phosphoinositide conversion in endocytosis and the endolysosomal system.* J Biol Chem, 2018. **293**(5): p. 1526-1535.
24. Gillooly, D.J., A. Simonsen, and H. Stenmark, *Phosphoinositides and phagocytosis.* J Cell Biol, 2001. **155**(1): p. 15-7.
25. Botelho, R.J., C.C. Scott, and S. Grinstein, *Phosphoinositide involvement in phagocytosis and phagosome maturation.* Curr Top Microbiol Immunol, 2004. **282**: p. 1-30.
26. Wang, Y., et al., *Chlamydial Histones Control Developmental Fitness in the Next Infection Cycle.* bioRxiv, 2026: p. 2026.02.10.705219.

REVIEWERS' COMMENTS

Reviewer #1 (Remarks to the Author):

General Comment
I have carefully examined the revised manuscript and found that, although most of my major and minor comments were adequately addressed, a few of the most critical concerns remain unresolved. In particular, it is still unclear whether the proposed model accurately reflects the intracellular behavior and functions of natural sphingomyelin (SM). I appreciate that there are technical limitations that may have prevented the authors from fully addressing these concerns. Nevertheless, because I believe this study has the potential to make a significant scientific contribution, I encourage the authors to further improve the manuscript by addressing the remaining issues outlined below.

Major Comments
1. L190–200: pGk12a, a palmitoylated peptide carrying positive charges, is a membrane probe that presumably interacts with acidic phospholipids such as phosphatidylserine and phosphoinositides. Therefore, the new data obtained using pGk12a do not provide evidence for the enriched distribution of natural SM in the EB nucleoid. Instead, the authors should have employed SM-specific probes, such as equinatoxin II-derived probes (e.g., PMID: 41267463; 40101206), in a post-expansion staining protocol. Throughout the manuscript, the authors should carefully avoid implying that the behavior of the unnatural SM analogs reflects that of natural SM unless direct and compelling evidence for the enriched distribution of natural SM is provided.

To our knowledge, it is unclear whether pGK13a preferentially binds to certain lipid species. In theory, the fixation process could also alter the charge of lipid species. However, we agree with the reviewer that because of the positive charge of pGK13a, a preference for rather negatively charged lipids is likely. We included this aspect in the discussion of the revised manuscript (line 345-347, revised manuscript).

Instead of using equinatoxin II as suggested by the reviewer, we had previously explored an alternative sphingomyelin-specific probe based on the C-terminal domain of Lysenin fused to GFP (Lysenin^{CTD}-GFP) (PMID: 32649908), which was kindly provided by the lab of Felix Randow (MRC LMB Cambridge, UK). Without permeabilization, Lysenin^{CTD}-GFP produced strong plasma membrane staining, as the protein probe cannot diffuse into the fixed cell. When we permeabilized the cells (with TX100 or saponin), only weak and miss localized staining was observed, appearing as aggregates rather than labeling cellular membranes. We therefore concluded that this approach is not suitable for staining intracellular sphingomyelin-containing membranes/inclusions in our experimental system.

2. L273–274: "BODIPY-FL-C12-SM, a lipid analogue that unlike TFSMs possess a naturally, even though rarely occurring, acyl chain length and hence, should adequately mimic the

physiological lipid transport." I do not agree with this argument. Although BODIPY-FL-C12-SM may be more hydrophobic than the TFSM analogs, it is still considerably less hydrophobic than C16-SM, a representative natural SM species.

We agree with the reviewer and changed the statement accordingly. It now reads (line 225-228, revised manuscript):

"Therefore, we treated infected HeLa cells with BODIPY-FL-C_{12}-SM, a lipid analogue that possesses a longer acyl chain than TFSMs. Hence, it should more adequately mimic the physiological transport of naturally occurring lipids. However, it is still less hydrophobic than naturally occurring SM species such as C_{16}-SM and thus may behave differently."

3. L369–371: " These observations support a model in which TFSM is transiently metabolized to ceramide, that subsequently serves as a substrate for TFSM1-SM synthesis, resulting in the accumulation of TFSM1-SM in the inclusion." I agree with this interpretation. Although the FRET-based data presented in Fig. 5 and Supplementary Fig. 6 strongly suggest that TFSM is metabolized in infected host cells, the finding that the FRET changes are independent of host SMases or CERT does not, by itself, provide compelling evidence that the conversion of TFSMs to their corresponding ceramide analogs is independent of these host factors. Therefore, the authors should clearly state whether the conversion of TFSMs to their ceramide analogs is independent of host SMases (acid SMase and neutral SMases). If so, the authors should briefly discuss the possibility that TFSMs are metabolized by chlamydial enzymes. Conversely, if the conversion is dependent on host SMases, the authors should discuss the possibility that host SMase(s), together with CERT, contribute to the delivery of natural ceramides to the inclusion for endogenous SM synthesis. In the latter case, Fig. 7 should also be revised to reflect this possibility.

We thank the reviewer for this important point. We agree that our FRET assay does not directly measure the enzymatic conversion of TFSM into its corresponding ceramide analog. Rather, it reports the relative abundance of intact versus metabolized fluorescent lipids within the analyzed region. Consequently, the absence of an effect in ASM-deficient cells, upon NSM inhibition, or in CERT-deficient cells does not allow us to conclude that these host factors are dispensable for the initial hydrolysis step. Instead, our data demonstrate that the overall accumulation of metabolized TFSM within chlamydial inclusions is not detectably altered under these conditions.

We have revised the text accordingly to avoid overstating this conclusion (line 278-280, revised manuscript):

"However, the enrichment of metabolized TFSM within inclusions was still detected in CERT K.O. cells by the FRET-based readout, even though with slightly delayed kinetic, supporting that metabolized TFSM accumulates within the inclusion even in the absence of detectable contributions from host ASM and NSM activity, or CERT under our experimental conditions."

Minor Comments

4. L93: "sphingomyelin" can be replaced with the abbreviation "SM."

We changed “sphingomyelin” to SM. (line 91, revised manuscript)

5. L93: "SM distribution" should be revised to, for example, "distribution of the SM derivatives" (see also Major Comment 1).

We agree and changed the sentence to: (line 91-92)

“We previously introduced two trifunctional SM derivatives (TFSM1 and TFSM2) that can be visualized at nanoscale resolution via 4x ExM **(Supp. Fig. 1, Supp. Note 1**).”

6. L109–110: "...detected a similarly localized accumulation to that observed in TFSM2-labeled samples (Fig. 1d), indicating that one pole of the EB is enriched in both sphingolipids and proteins" should be toned down. For example, replacing "indicating" with "suggesting" would be more appropriate (see also Major Comment 1).

We replaced “indicating” with “suggesting”.

7. L153: "globe-shaped TFSM-enriched structure": If the authors are referring to the blue-colored nucleoid region in Fig. 3b, it appears more dumpling-shaped or rugby ball-shaped (at least to me) rather than globe-shaped.

We believe that the reviewer refers to line L160 (manuscript from previous submission). There we refer to Fig. 3, d, e (ExM) and not Fig. 3 b (cryo-ET). However, we agree with the reviewer that nucleoids/TFSM accumulations are not perfectly globe-shaped. We changed all instances of “globe-shape” to “spheroidal”.

Reviewer #2 (Remarks to the Author):

Thank you for taking the time to address my comments. I don't have any further concerns.
